# OptiMo‐LDLr: An Integrated In Silico Model with Enhanced Predictive Power for LDL Receptor Variants, Unraveling Hot Spot Pathogenic Residues

**DOI:** 10.1002/advs.202305177

**Published:** 2024-01-23

**Authors:** Asier Larrea‐Sebal, Iñaki Sasiain, Shifa Jebari‐Benslaiman, Unai Galicia‐Garcia, Kepa B. Uribe, Asier Benito‐Vicente, Irene Gracia‐Rubio, Harbil Bediaga‐Bañeres, Sonia Arrasate, Ana Cenarro, Fernando Civeira, Humberto González‐Díaz, Cesar Martín

**Affiliations:** ^1^ Biofisika Institute (UPV/EHU, CSIC) Barrio Sarriena s/n. Leioa Bizkaia 48940 Spain; ^2^ Department of Biochemistry and Molecular Biology Universidad del País Vasco UPV/EHU Leioa Bizkaia 48940 Spain; ^3^ Fundación Biofisika Bizkaia Barrio Sarriena s/n. Leioa Bizkaia 48940 Spain; ^4^ Lipid Unit, Hospital Universitario Miguel Servet, IIS Aragon, CIBERCV Universidad de Zaragoza Zaragoza 50009 Spain; ^5^ Department of Physical Chemistry University of Basque Country UPV/EHU Leioa 48940 Spain; ^6^ Department of Organic and Chemistry University of the Basque Country UPV/EHU Leioa 48940 Spain; ^7^ Ikerbasque, Basque Foundation for Science Bilbao Bizkaia 48013 Spain

**Keywords:** hot spot, in silico, LDLr, predictive software

## Abstract

Familial hypercholesterolemia (FH) is an inherited metabolic disease affecting cholesterol metabolism, with 90% of cases caused by mutations in the *LDL receptor* gene (*LDLR*), primarily missense mutations. This study aims to integrate six commonly used predictive software to create a new model for predicting *LDLR* mutation pathogenicity and mapping hot spot residues. Six predictive‐software are selected: Polyphen‐2, SIFT, MutationTaster, REVEL, VARITY, and MLb‐LDLr. Software accuracy is tested with the characterized variants annotated in ClinVar and, by bioinformatic and machine learning techniques all models are integrated into a more accurate one. The resulting optimized model presents a specificity of 96.71% and a sensitivity of 98.36%. Hot spot residues with high potential of pathogenicity appear across all domains except for the signal peptide and the O‐linked domain. In addition, translating this information into 3D structure of the LDLr highlights potentially pathogenic clusters within the different domains, which may be related to specific biological function. The results of this work provide a powerful tool to classify *LDLR* pathogenic variants. Moreover, an open‐access guide user interface (OptiMo‐LDLr) is provided to the scientific community. This study shows that combination of several predictive software results in a more accurate prediction to help clinicians in FH diagnosis.

## Graphical abstract

OptiMo‐LDLr is an advanced computational model that improves predictions of LDL receptor (LDLr) variant pathogenicity. It outperforms other methods, accurately distinguishing between pathogenic and benign LDLr variants, and identifies critical residues in all domains except the signal peptide and O‐linked domain. OptiMo‐LDLr also visualizes 3D hot spot areas, enhancing understanding of FH and aiding clinicians in managing this genetic condition.

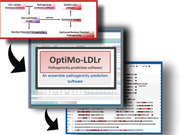

## 1 Introduction

Familial hypercholesterolemia (FH) is a common inherited metabolic disease causing the malfunction of cholesterol metabolism^[^ ^1^ ^]^ with a prevalence between 1:200 and 1:250 in several populations.^[^ ^2^ ^]^ In >90% of the cases, FH is caused by mutations in the *LDL receptor* gene (*LDLR*), being missense mutations the most common ones.^[^ ^3^ ^]^ In fact, >1300 mutations causing amino acid substitution have already been reported in ClinVar, a freely accessible public archive reporting disease‐related genomic variations.^[^ ^4^ ^]^ Missense mutations in the *LDLR* have different impact on protein activity depending on the protein's domain they locate. Hence, mutations residing in functionally or structurally relevant regions are more susceptible of being pathogenic.^[^ ^5^ ^]^ The LDL receptor (LDLr) is a trans‐membrane protein involved in plasma LDL cholesterol (LDL‐c) clearance, a process that occurs upon binding of LDLr to apolipoprotein B‐100 (ApoB‐100) a main component of the LDL particle.^[^ ^6^ ^]^ LDLr consists of 860 amino acids organized in nine different domains as follows: signal peptide (SP), LDL binding domain (LBD), epidermal growth factor like domain A (EGF‐A), epidermal growth factor like domain B (EGF‐B), β‐propeller, epidermal growth factor‐like domain C (EGF‐C), Oxygen‐linked glycosylation domain (O‐linked), transmembrane domain and the cytosolic domain.^[^ ^6^ ^]^

The different impact on the biological effect caused by the amino acid substitutions in the LDLr makes essential determining their pathogenicity to facilitate definitive diagnosis and to gain information about the cardiovascular risk of each variant.^[^ ^7^ ^]^ In addition to traditional cascade screening and in vitro assessment of the activity of genetic variants, computational predictive software has arisen as a powerful tool to predict the pathogenicity of *LDLR* variants;^[^ ^8^ ^]^ among them, Polyphen‐2 (Polymorphism Phenotyping v2),^[^ ^9^ ^]^ MutationTaster,^[^ ^10^ ^]^ SIFT (Sorting Intolerant From Tolerant)^[^ ^11^ ^]^ REVEL (Rare Exome Variant Ensemble Learner)^[^ ^12^ ^]^ and VARITY.^[^ ^13^ ^]^ Very recently, more specific software designed for *LDLR*, such as MLb‐LDLr (Machine Learning based low‐density lipoprotein receptor software) has been developed^[^ ^14^ ^]^ or SFIP‐MutID (Structure‐based functional impact prediction for mutation identification).^[^ ^15^ ^]^ This seven software represent the state of the art in 2023, providing comprehensive and advanced tools for predicting the pathogenicity of *LDLR* variants.

Currently, discrepancies arise in the results provided by individual software programs due to variations in the criteria they employ to infer the pathogenicity of substitutions, therefore collective analysis of predictions from multiple software tools is frequently undertaken to increase overall predictive efficacy. To enhance the prediction of pathogenic variants in the *LDLR*, this study aims to integrate the predicted effects of each missense mutation using existing software, thereby enhancing the individual predictive capabilities of these tools. The optimized model, integrated into the OptiMo‐LDLr software, enhances the predictive capacity for pathogenicity of LDLr variants. The results of this work not only provide a powerful tool for classifying *LDLR* pathogenic variants but also contribute to deciphering pathogenic hot spots within the receptor. This information is further translated to the 3D structure of the LDLr, elucidating potentially pathogenic regions within different domains that may be associated with specific biological functions. Additionally, an open‐access guide user interface (https://www.ehu.eus/es/web/hypercholesterolemia‐mechanisms/optimo‐ldlr) is made available to the scientific community.

## 2 Experimental Section

### 2.1 Predictive Software

The human wild‐type (wt) LDLr amino acid sequence (Uniprot #P01130) was used as template.

Six software that predict the pathogenicity caused by amino acid substitution were selected, those not specifically dedicated to LDLr (PolyPhen‐2, SIFT, MutationTaster, REVEL and VARITY) and one specifically developed for LDLr (MLb‐LDLr). Each software uses different databases and methodologies for predicting pathogenicity. Concerning the general prediction software, PolyPhen‐2 (http://genetics.bwh.harvard.edu/pph2/) combines sequence‐ and structure‐based approaches,^[^ ^9^ ^]^ SIFT (https://sift.bii.a‐star.edu.sg/index.html) infers pathogenicity from sequence similarity,^[^ ^11^ ^]^ MutationTaster (https://www.mutationtaster.org/) is based on evolutionary conservation, splice‐site changes, protein's features loss and changes that might affect mRNA expression level,^[^ ^10^ ^]^ REVEL (https://sites.google.com/site/revelgenomics/) incorporates many predictive tools, but is trained on recently discovered variants not used by them^[^ ^12^ ^]^ and VARITY (http://varity.varianteffect.org/) has been optimized to predict the pathogenicity of rare missense variants.^[^ ^13^ ^]^ Regarding the specific software for *LDLR*, MLb‐LDLr (https://www.ehu.eus/en/web/hypercholesterolemia‐mechanisms/mlb‐ldlr1) considers several factors such as conservation, physicochemical characteristics of amino acids and structural features of LDLr.^[^ ^14^ ^]^ SFIP‐MutID, a predictive software specifically designed for *LDLR* ^[^ ^15^ ^]^ was not included in the ensemble model developed in this study due to its limited predictive capacity.

### 2.2 Database

In addition, the public database of human genetic variants and their significance to disease (ClinVar),^[^ ^16^ ^]^ managed by the National Centre for Biotechnology Information (NCBI), was used as a source for gathering information related to the *LDLR* variants described so far. ClinVar database categorizes *LDLR* variants into six subtypes attending the type of mutation: frameshift, missense, nonsense, splice site, noncoding RNA, and untranslated region (UTR). However, the biological effect of most of these subtypes could be easily predicted without the use of any predicting tool. Frameshift, nonsense, and splice site variants were mostly pathogenic, and the only variable was the position in which the mutation occurs. On the other hand, noncoding RNA and UTR variants did not affect the protein directly, so their effects were mostly benign. Among all mutation types, missense mutations were the most appealing ones to map hot spots within the LDLr sequence. Altering a single amino acid, as in the site‐directed mutagenesis analysis, the biological significance of that residue could be inferred. Therefore, missense mutations were selected to perform a hot spot amino acid analysis within LDLr due to the relevance of this kind of mutations on protein activity. Out of 1475 missense variants annotated in ClinVar (Last update: July 18th, 2023) a datasheet containing 669 was obtained after excluding variants without clinical significance and those appearing in more than one subtype.90% of the obtained variants were classified as pathogenic or likely pathogenic and 10% as benign or likely benign.^[^ ^4^ ^]^ Missense variants classified as Pathogenic/Likely Pathogenic or Benign/Likely Benign were grouped, making not distinction among them throughout this work.

### 2.3 Data Acquisition, Homogenization, Normalization, and Combination

First, the scores (*B*) provided by each software (*s*) for all the substitutions were obtained. Although some software provides pathogenicity predictions for all possible mutations for each residue (19 mutations per residue), REVEL and VARITY only provide predictions for the most probable variants, those in which a single nucleotide was modified. As all the mutations in ClinVar belong to this group, it was next decided to only analyze the most probable mutations, those obtained due to the modification of a single nucleotide. This means that the number of probable mutations per residue varies depending on the amino acid.

PolyPhen‐2 and SIFT provide the option of batch querying, while MutationTaster and MLb‐LDLr only allow for individual queries. A script was written to automatize data acquisition for MutationTaster using Python (v.3.10.4). MLb‐LDLr prediction data was obtained from the information published previously.^[^ ^14^ ^]^ In the case of REVEL and VARITY, predictions for all probable mutation have already been made, and are readily available for download.

Acquisition of pathogenicity score for some substitutions (mainly the ones located in splicing sites) was not possible in MutationTaster and therefore, to get comparable scores the obtained data was homogenized and normalized as follows: the format of the obtained data was homogenized without altering its meaning, calculating the probability of each substitution (*i*) being pathogenic (*P* = 1) according to each software (*s*). That value was denominated *p*(*P_si_* = 1) in which 1 is considered as the most pathogenic value and 0 as the least. The scores provided by PolyPhen‐2, REVEL, and VARITY already meet the previous conditions, so no adaptation was needed. MutationTaster and MLb‐LDLr provide the probability of each substitution to be benign or pathogenic according to the most likely effect. Therefore, the probability of a substitution to be benign is provided *p*(*P_si_* = 0), instead of pathogenic, *p*(*P_si_* = 1), when *p*(*P_si_* = 1) < *t_s_*, considering *t_s_* as the threshold between classifying a substitution as benign or pathogenic for each software. In order to homogenize the scores, *p*(*P_si_* = 1) was calculated for all the substitutions when *p*(*P_si_* = 0) was provided following Equation (1). Regarding the data given by SIFT, values between 0 and 0.05 are considered as pathogenic, and the ones >0.05 as benign. This score had also to be adapted to establish 1 as maximum pathogenicity value and 0 as minimum, similarly to the rest of the software, following Equation (2).

(1) $$p\left(P_{si}=1\right)=1-p\left(P_{si}=0\right)$$

(2) $$p\left(P_{SIFT,i}=1\right)=1-B_{SIFT}$$

Then, all the data from the different software was normalized, rescaling them between 0 (most benign value) and 1 (most pathogenic value) considering 0.5 as the threshold. Therefore, a linear mathematical expression which does not perturb the original meanings was used (Equation (3)) where *p*(*P_si_* = 1) represents the previously homogenized score for each software and substitution, *max*[*p*(*P_si_* = 1)] its maximum value (equal to 1 in all the software) and *min*[*p*(*P_si_* = 1)] its minimum value (equal to 0 in all the software); *t_s_* is the threshold between considering a substitution as benign or pathogenic in the original scale (which can be also interpreted as the minimum value of the substitutions predicted as pathogenic and the maximum of the ones predicted as benign), and *p*(*P_si_* = 1)_*Nor*_ represents the normalized probability for each software and substitution. The values equal to the threshold were considered as pathogenic, including them in the first sub equation of Equation (2), as SIFT, the only model that provides those, considers them so.

(3) $$\begin{array}{c} p{\left(P_{si}=1\right)}_{Nor}=\left\{\begin{array}{c} p\left(P_{si}=1\right)\geq t_{s};\;\frac{p\left(P_{si}=1\right)-t_{s}}{max\left[p(P_{si}=1)\right]-t_{s}}\\ p\left(P_{si}=1\right)<t_{s};\;\frac{p\left(P_{si}=1\right)-min\left[p(P_{si}=1)\right]}{t_{s}-min\left[p(P_{si}=1)\right]} \end{array}\right. \end{array}$$

After normalization, the new (*p*(*P_si_* = 1)_*Nor*_) values were combined creating a new score that represents the overall predicted pathogenicity of each substitution, *f*(*P_i_*)_*calc*_, between 0 (most benign value) and 4 (most pathogenic value), considering 2 as the threshold. *f*(*P_i_*)_*calc*_ values were calculated according to Equation (4). The combined score of the substitutions in which the data from MutationTaster was missing, was rescaled by multiplying it by $\frac{6}{5}$ to avoid their misinterpretation.

(4) $$f{\left(P_{i}\right)}_{calc}=\sum_{s\;=\;1}^{s\;=\;6}p{\left(P_{si}=1\right)}_{Nor}$$

### 2.4 Pathogenicity of Amino Acid Substitutions: Statistical Analysis

The normalized pathogenicity score predicted by each software, *p*(*P_si_* = 1)_*Nor*_, was compared by determining the standard deviation between the obtained values and calculating the divergence in the predictions made by each software. Besides, specificity and sensitivity were calculated with the already characterized *LDLR* substitutions in ClinVar for each software. After performing the combination of the normalized scores, *f*(*P_i_*)_*calc*_, specificity and sensitivity were also determined for the new score with the already characterized substitutions in ClinVar.

### 2.5 Potential Pathogenicity of Residues

To determine the biological importance of each residue, the potential pathogenicity (*PP*) function was calculated for each residue (*r*) and software (*s*), *f*(*PP_rs_*). High potential pathogenicity values correspond to residues whose probable substitutions were mostly predicted as pathogenic. Hence, they were interpreted as biologically relevant for the function of LDLr. Starting from the normalized scores of each software, *p*(*P_si_* = 1)_*Nor*_, the potential pathogenicity was determined by calculating the average value of the probable substitutions for each residue and software, *f*(*PP_rs_*)_*av*_, according to Equation (5). This way, a value between 0 (low importance) and 1 (high importance) was obtained, considering 0.5 as the threshold. In Equation (5), *n_sr_* represents the number of substitutions in which pathogenicity scores are provided for each residue in each software. The calculated values by each software were then combined according to Equation (6) creating a new score system, *f*(*PP_r_*)_*calc*_, between 0 (low importance) and 7 (high importance) in which 3.5 is considered the threshold value. This represents the overall predicted potential pathogenicity of each residue.

(5) $$f{\left(PP_{rs}\right)}_{av}=\frac{1}{n_{sr}}\times \sum_{i\;=\;1}^{i\;=n_{sr}}p{\left(P_{si}=1\right)}_{Nor}$$

(6) $$f{\left(PP_{r}\right)}_{calc}=\sum_{s=1}^{s=6}f{\left(PP_{rs}\right)}_{av}$$

### 2.6 Optimization of the Software Combination

The calculated software combination (*f*(*P_i_*)_*calc*_) was optimized by modifying the weight of each software in the final score. Therefore, coefficients for each software (e) were optimised using Excel Solver Evolutionary Algorithm (ESEA) (detailed in the Supporting Information).^[^ ^17^ ^]^ The threshold (*e_s_*) that defines the minimum pathogenicity value of a pathogenic variant was also used as an optimization variable. The optimization coefficients were estimated by maximizing *F*_0_, which was determined according to Equation (7) using the data available in ClinVar. This allowed obtaining a balanced tool able to accurately predict both pathogenic and benign substitutions.

(7) $$F_{0}=Specificity\times Sensitivity$$

The optimized score that represents the overall predicted pathogenicity of each substitution (*f*(*P_i_*)_*opt*_) was determined according to Equation (8). In the cases where data from MutationTaster was missing the optimized combination scores of the substitutions were rescaled by multiplying it by $\frac{6}{\left(6-e_{MutT}\right)}$ to avoid their misinterpretation.

(8) $$f{\left(P_{i}\right)}_{opt}=e_{t}-\sum_{s\;=\;1}^{s\;=\;6}e_{s}\times p{\left(P_{si}=1\right)}_{Nor}$$

The optimization coefficients were also used for the calculation of the optimized potential pathogenicity values (*f*(*PP_r_*)_*opt*_) for each residue. These were applied to weight the combination of the potential pathogenicity values determined for each software (*f*(*PP_rs_*)_*av*_) that had been previously calculated through Equation (3). The optimized potential pathogenicity was calculated according to Equation (9).

(9) $$f{\left(PP_{r}\right)}_{opt}=e_{t}-\sum_{s\;=\;1}^{s\;=\;6}e_{s}\times f{\left(PP_{rs}\right)}_{av}$$

### 2.7 OptiMo‐LDLr Model Verification

In order to obtain an unbiased model, the database was divided into two groups: training (T) and validation (V). Variants in training group were used to optimize the parameters (*e_s_* and the threshold that divides benign and pathogenic variants) of the model. Afterward, those parameters were used with the validation group. This division prevents the creation of an over trained model, where its accuracy was overestimated. The variants were randomly assigned to training or validation series. Three‐quarters of the pathogenic variants (*n* = 457) were used for training and the remaining (*n* = 152) to validate the model. In the case of benign variants, two‐thirds (*n* = 40) were used in the training group, and the remaining (*n* = 20) were used in the validation group due to the limited number of annotated variants.

In addition, random bootstrapping training and validation subsets of the same sample size with replacement were used to test the sampling distribution. One thousand bootstrapped samples were tested, and sensitivity and specificity values were presented with 95% confidence intervals.

**Figure 1:**
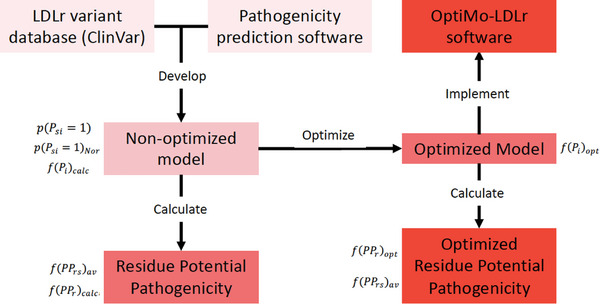
OptiMo‐LDLr flowchart. Pathogenicity predictions of six software were applied to 669 *LDLR* variants with known pathogenicity obtained from ClinVar. A non‐optimized predictive model was developed using the predictions of the 6 software, and the potential pathogenicity of each residue in LDLr was calculated. Afterward, the model was optimized using ESEA algorithm to increase the accuracy of the predictions. The pathogenicity predictions of the resulting model were implemented into a user‐friendly software. The potential pathogenicity of each LDLr residue were calculated using the optimized model, and the results were displayed in a hot spot map.

A detailed flowchart illustrating the step‐by‐step development of the OptiMo‐LDLr predictive model is shown in **Figure** **1**.

### 2.8 Identification of Pathogenic Hot Spots

The obtained optimized combination of the potential pathogenicity was used to map pathogenic hot spots of LDLr. To achieve this objective, the residues of LDLr were categorized based on their estimated potential pathogenicity values. Those with values below 4.5 were represented in white, while those falling within the ranges of (4.5–4.9), (4.9–5.3), (5.3–5.7), (5.7–6.1), and (6.1–6.5) including the upper limit were depicted using varying intensities of red. The most intense shade of red was used to indicate the highest potential pathogenicity values. The resulting hot spot map was then analyzed and interpreted using existing bibliographical references about the structure and function of LDLr and data available in ClinVar.

### 2.9 Hot spot 3D Mapping

For the 3D mapping of LDLr hot spot regions, the crystallographic structure of LDLr was obtained from PDB (Experimental PDB structure: 1N7D) and the software Pymol (The PyMOL Molecular Graphics System, Version 2.0 Schrödinger, LLC) was used for colouring the residues.

## 3 Results

### 3.1 Combination of the Normalized Data Improves its Predicting Skills

**Figure 2:**
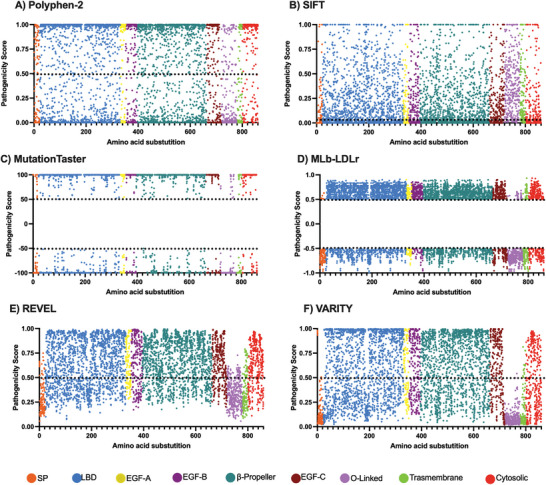
Original pathogenicity scores provided by A) Polyphen‐2, B) SIFT, C) MutationTaster, D) MLb‐LDLr, E) REVEL, and F) VARITY. Each point represents the scores provided by each software. The black dotted line is the threshold between considering a substitution as benign or pathogenic. In (A,B,E,F) values above the threshold are considered pathogenic and the ones below the threshold, benign. In (C) values above 50 represent the probability of being pathogenic and below −50 of being benign. In (D) values above 0.5 represent the probability of being pathogenic and below −0.5 of being benign. SP: Signal Peptide; LBD: Ligand Binding Domain; EGF: Epidermal Growth Factor; O‐linked: Oxygen‐linked glycosylation domain.

First, the pathogenicity scores provided by Polyphen‐2, MutationTaster, SIFT, MLb‐LDLr, REVEL and VARITY were obtained (**Figure** **2**). Polyphen‐2, REVEL and VARITY provide the data in a scale between 0 (the most benign value) and 1 (the most pathogenic value), establishing the threshold in 0.5 for Polyphen‐2 and VARITY (Figure 2A,F). Given that REVEL does not inherently specify a threshold value, we established a threshold of 0.5 for REVEL in our analysis (Figure 2E). For SIFT (Figure 2B), the threshold is defined as 0.05, where 0 represents the most pathogenic value and 1 the most benign. In contrast, MutationTaster (Figure 2C) and MLb‐LDLr (Figure 2D) offer the probability of each substitution being benign or pathogenic, rather than directly indicating pathogenicity. Consequently, MutationTaster or MLb‐LDLr assign values above 50 and 0.5 to substitutions considered pathogenic and values below −50 and −0.5 to those deemed benign, respectively.

**Figure 3:**
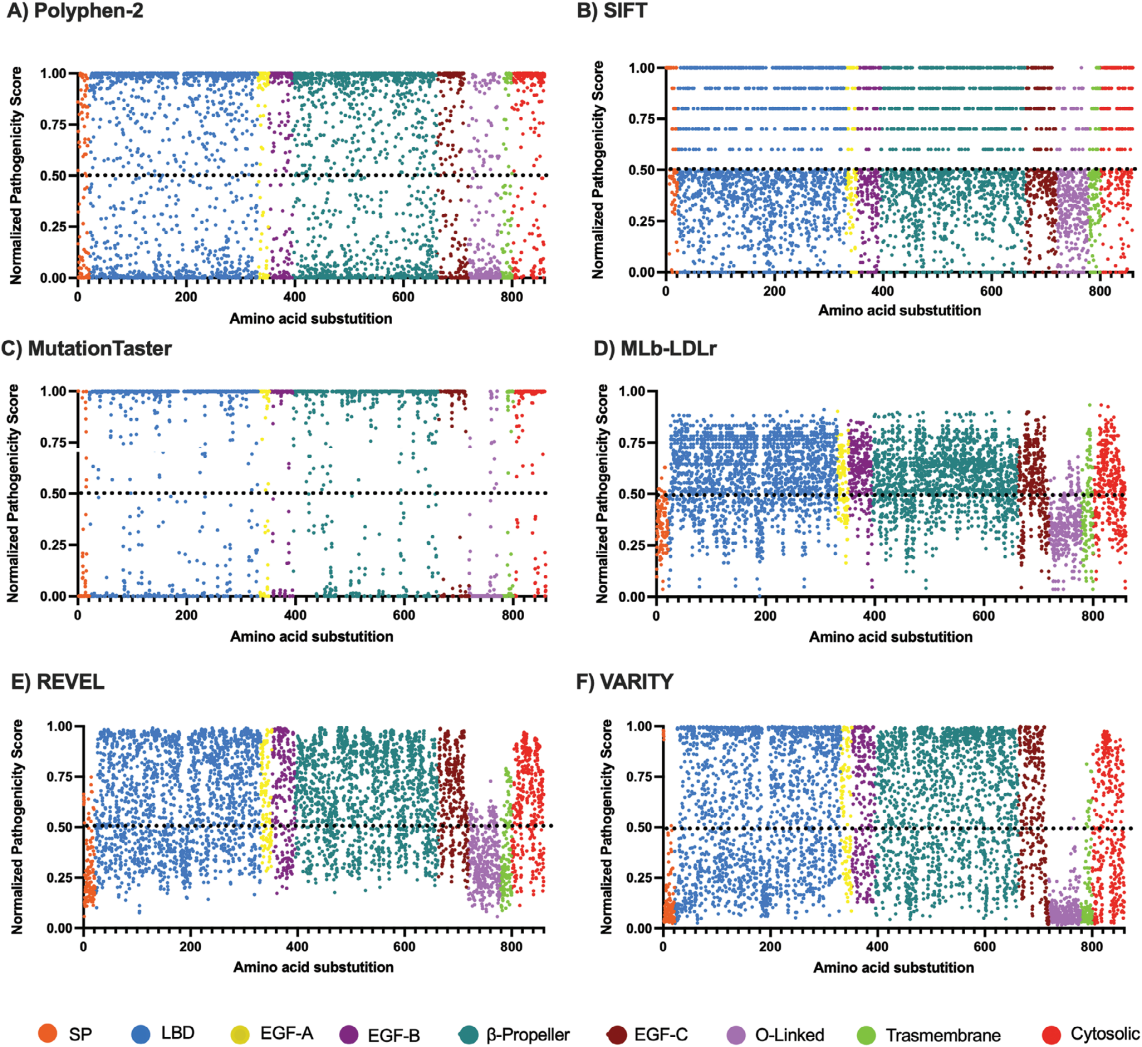
Normalized scores of A) Polyphen‐2, B) SIFT, C) MutationTaster, D) MLb‐LDLr, E) REVEL, and F) VARITY. The original scores were rescaled using Equation (3) to obtain comparable values. The black dotted line represents the threshold between considering a substitution as benign, when its score is below it, or pathogenic, when it is above it. SP: Signal Peptide; LBD: Ligand Binding Domain; EGF: Epidermal Growth Factor; O‐linked: Oxygen‐linked glycosylation domain.

The described data was then rescaled (between 0 as the most benign value, and 1, the most pathogenic value, considering 0.5 as the threshold) aiming to obtain comparable values. The pathogenicity probability estimated by Polyphen‐2, REVEL, and VARITY (**Figure** **3**A,E,F) remain constant, as the new normalized scale is equal to its original one. Besides, its scores are equally distributed on the new scale. Regarding SIFT (Figure 3B), the substitutions over the threshold appear scaled, due to fact that the values of the predicted pathogenic substitutions in the original scale were scored between 0 and 0.05, having only two decimal places. The scores are also equally distributed on the new scale. On the other hand, the pathogenicity probabilities estimated by MutationTaster (Figure 3C) are polarized, being the majority in the most extreme values, and MLb‐LDLr (Figure 3D) shows the opposite phenomena, as the scores calculated are located close to the threshold.

**Figure 4:**
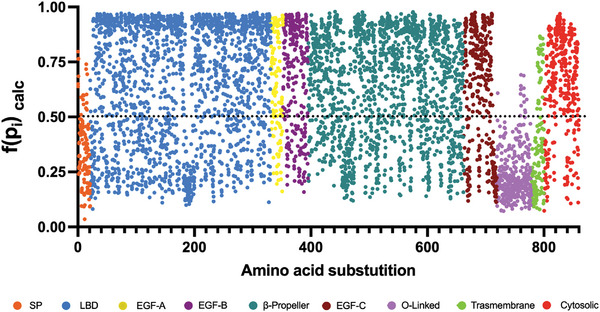
Non‐optimized combination of the normalized scores. The normalized scores from the different software were combined using Equation (4). Black dotted line represents the threshold between considering a substitution as benign (scores below it), or pathogenic (scores above it). SP: Signal Peptide; LBD: Ligand Binding Domain; EGF: Epidermal Growth Factor; O‐linked: Oxygen‐linked glycosylation domain.

In order to integrate the different predictive software, we next combined the normalized data (**Figure** **4**) thus allowing to integrate the different pathogenicity prediction strategies of each software. As shown in Figure 4, it is noticeable that the graphical representation of the combination shows a high dependency on the LDLr domains, as it had been previously observed in the results of some software, specially MLb‐LDLr, REVEL and VARITY, whose graphical representation (Figure 3D,E,F) shows higher similarity to the software combination.

### 3.2 Potential Pathogenicity of Residues

**Figure 5:**
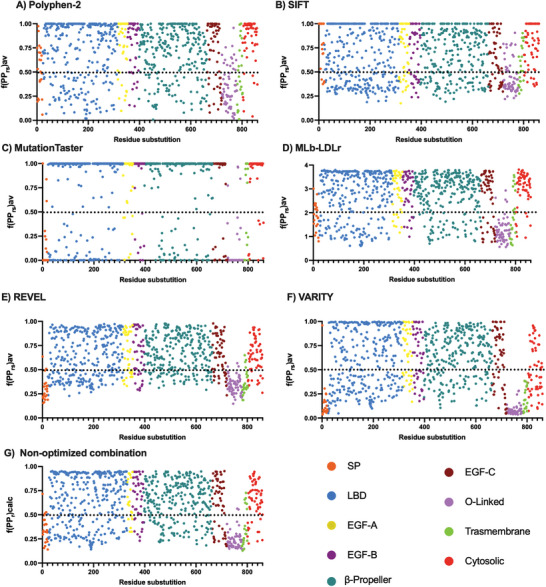
Potential pathogenicity of LDLr obtained from the predicted pathogenicity score of A) Polyphen‐2, B) SIFT, C) MutationTaster, D) MLb‐LDLr, E) REVEL, F) VARITY, and G) their non‐optimized combination. The potential pathogenicity value is the average of each residue`s probable substitutions’ normalized score and represents the biological transcendence of each. The black dotted line represents the threshold between considering a substitution as benign, when its score is under it, or pathogenic, when the score is above it. SP: Signal Peptide; LBD: Ligand Binding Domain; EGF: Epidermal Growth Factor; O‐linked: Oxygen‐linked glycosylation domain.

The potential pathogenicity for each residue and each software was estimated as described in Experimental Section. The potential pathogenicity in Polyphen‐2 (**Figure** **5**A), MutationTaster (Figure 5C), MLb‐LDLr (Figure 5D), REVEL (Figure 5E), and VARITY (Figure 5F) show high similarity to the rescaled mutations (Figure 3A,C–F), but with a lower density of scores. Concerning SIFT (Figure 5B), the previously described escalation of the scores (Figure 3B) disappears as the 4–7 probable substitutions for each residue have been considered to calculate the potential pathogenicity. The values obtained from each software were also combined as described in Materials and Methods. As shown in Figure 5F, the potential pathogenicity shows similar distribution pattern than the pathogenicity predictions (Figure 4).

### 3.3 Optimization of Software Combination Improves its Predictive Skills

Finally, the combination of the substitutions’ pathogenicity score and of the potential pathogenicity were optimized. The optimization coefficients were estimated using ESEA to weight the contribution of each software and the threshold, and the final model is shown in Equation (10).

(10) $$\begin{array}{ccc} f{\left(P_{i}\right)}_{opt} & = & -3.43+0.76*{\left(P_{SIFT}=1\right)}_{Nor}+1.38*{\left(P_{PolyPhen2}=1\right)}_{Nor}\\ & & +\;0.67*\;{\left(P_{MLb-LDLr}=1\right)}_{Nor}+0.91*\;{\left(P_{MutationTaster}=1\right)}_{Nor}\\ & & +\;0.51*{\left(P_{REVEL}=1\right)}_{Nor}+1.82*{\left(P_{VERITY}=1\right)}_{Nor} \end{array}$$

where N_Training_ = 497, N_Validation_ = 172, N_Total_ = 669, 𝝌2 = 452 and P<0.05. The model classifies correctly 96.5% of pathogenic variants (440 of 456) and 97.5% of benign variants (40 of 41) on training, and 97.4% of pathogenic (149 of 153) and 100.0% of benign (20 of 20) on validation. Statistics of each software and model are shown in **Table** **1** and the prediction of each individual software is shown in Table S1 (Supporting Information).

**Table 1:** Accuracy of predictive software.

	Specificity [%]	Sensitivity [%]
SIFT	86.67	89.00
PolyPhen‐2	81.67	94.42
Mutation Taster	67.86	97.12
MLb‐LDLr	81.67	92.28
REVEL	36.66	97.37
VARITY	95.56	91.66
Non‐optimized	81.67	97.53
OptiMo‐LDLr	96.71	98.36

Regarding the predictive capacity of the software, nearly all sensitivity values are above 90%, while the specificity values are ≈80% with some exceptions. OptiMo‐LDLr is the most sensitive (98.36%) and specific (96.7%) model, as well as the most balanced one, due to the optimization process followed in Equation (7). Both MutationTaster and REVEL show the second‐best sensitivity values, but they are by far the least specific ones with 67.86% and 36.66%, respectively. In the case of REVEL, the observed low specificity might potentially be attributed to the threshold we established for the model, although it is not considered a significant factor affecting its overall contribution to the model. Regarding the non‐optimized model, the specificity value matches that obtained by Polyphen‐2 and MLb‐LDLr (Specificity = 81.67%). However, the sensitivity rate is enhanced, achieving the second‐best value (Sensitivity = 97.53%) (Table 1).

Variants annotated on ClinVar database were used to test the accuracy of the software. N_Pathogenic_ = 583, N_Benign_ = 58.

To test the validity of these results, a random bootstrapping resampling was performed to measure the effect of the starting dataset on the final results. The more similar the statistics are in both cases, the less the effect of the starting dataset. Sensitivity and specificity values of the predictive software and the combination before and after the bootstrapping process are shown in **Table** **2**.

**Table 2:** Model training, validation, and bootstrapping statistics.

Statistics	Non‐bootstrapped				Bootstrapped			
	T		V		T		V	
	Sp	Sn	Sp	Sn	Sp	Sn	Sp	Sn
SIFT	85.00	89.93	90.00	86.18	86.60	88.88	86.93	88.81
PolyPhen‐2	82.50	95.19	80.00	92.11	81.60	94.44	81.61	94.40
Mutation Taster	72.22	96.67	60.00	98.52	69.80	96.45	70.49	96.50
MLb‐LDLr	77.50	92.56	90.00	91.45	81.48	92.29	81.99	92.31
REVEL	37.34	96.89	34.53	98.52	36.89	95.62	35.34	98.23
VARITY	95.89	92.02	95.00	90.94	94.79	90.62	95.98	92.48
Non‐optimized	80.00	96.72	82.00	97.37	80.52	97.17	83.98	90.58
OptiMo‐LDLr	97.56	96.49	100	97.38	97.33	95.68	95.25	96.89

Statistics of the original sampling and randomly bootstrapped sampling. Variants annotated on ClinVar database were used to test the accuracy of the software. N_Training/pathogenic_ = 457 N_Training/benign_ = 40 N_Validation/pathogenic_ = 152 N_Validation/benign_ = 20. One thousand bootstrapped samples were used, and the results are shown with a 95% confidence interval. T = Training; V = Validation; Sn = Sensitivity; Sp = Specificity.

All statistics of non‐bootstrapped samples are within the 95% confidence interval obtained from bootstrapping, meaning that the sampling is unbiased. In addition, statistics in both training and validation groups are similar, even with the difference in the variant number.

**Figure 6:**
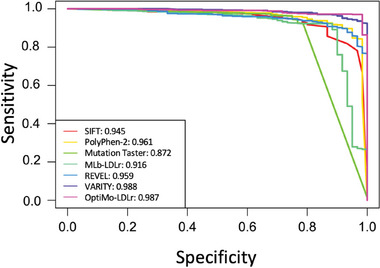
Performance of predictive software by Area Under Receiver Operating Curve (AUROC). AUROC test was carried out by modifying the threshold of each software and opposing sensitivity and specificity values.

Next, we performed the Area Under Receiver Operating Curve (AUROC) test to determine the model's ability to discriminate between positive and negative cases. It is obtained by modifying the threshold of the model, thus, its prediction of each variant. AUROC tests the performance of the model when different specificity and sensitivity values are needed. Hence, the higher the AUROC value, the more flexible the model is (**Figure** **6**).

AUROC values are given in percentages and models >80% are considered accurate. All the analyzed models demonstrate AUROC values exceeding 90%, indicating their reliability as predictive models. VARITY and OptiMo‐LDLr exhibit the highest scores, followed by PolyPhen‐2 and REVEL, respectively. While OptiMo‐LDLr significantly outperforms the others in terms of accuracy, the difference in AUROC values is not as substantial. This might be attributed to several factors. First, AUROC values are inherently large, with most software yielding AUROC values above 94%, leaving limited room for further improvement. In addition, the model is optimized for a specific threshold (the peak shown by OptiMo‐LDLr in Figure 6), although the bootstrapping results (Table 2) indicate that the accuracy does not decrease when using different variables for the model.

**Figure 7:**
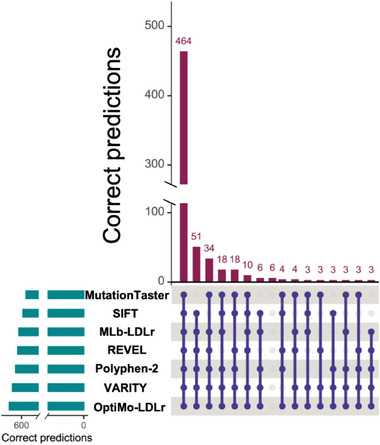
Visualization of comparative concordance of software using UpSet. This graph represents the overlaps and differences in the prediction of the software. Each set in the graph represents a unique combination of correct predictions, while the intersections indicate shared elements among the predictive software outputs. The smaller bars to the left indicate the number of correct predictions per software. Agreement between two software tools is not represented.

Additionally, we assessed the agreement among the predictive software. To facilitate a visual comparison of their concordance, we employed an UpSet graph (**Figure** **7**). This graphical representation provides an intuitive depiction of set intersections, highlighting both the overlaps and discrepancies in the predictions made by the different software tools. Each set within the graph represents a unique combination of correct predictions, and the intersections indicate shared elements among the predictive software outputs. The smaller bars situated to the left denote the number of correct predictions per software.

Figure 7 shows that there is a high level of agreement among all software tools for the majority of variants, with 464 out of 669 (69%) variants receiving unanimous agreement. Approximately 150 variants exhibit agreement among 5 to 6 software tools, while another 50 variants are predicted correctly by only 1 or 2 software tools. Remarkably, only 6 variants remain unpredicted by any of the software tools. Notably, there are no variants that are exclusively and correctly predicted solely by OptiMo‐LDLr.

**Figure 8:**
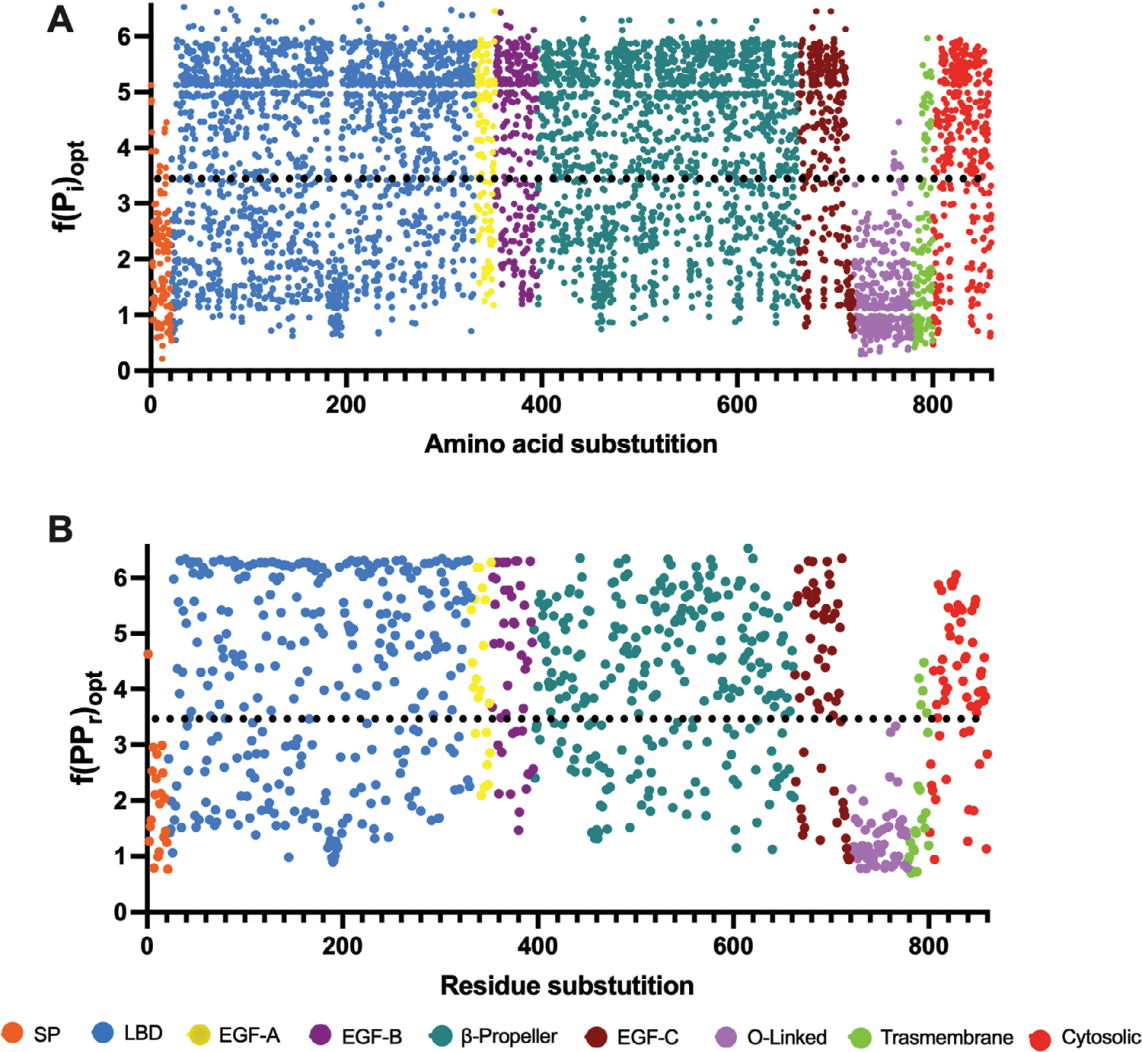
Optimized combination of A) normalized pathogenicity of substitutions and B) potential pathogenicity of residues. The optimization was performed using ESEA and Equation (8) for the optimized pathogenicity of substitutions, and Equation (9) for the optimized combination of potential pathogenicity, which represents the biological importance of each residue. The black dotted line represents the threshold between considering a substitution as benign, when its score is under it, or pathogenic, when the score is above it. SP: Signal Peptide; LBD: Ligand Binding Domain; EGF: Epidermal Growth Factor; O‐linked: Oxygen‐linked glycosylation domain.

Once demonstrated that the optimization model had better prediction abilities, we calculated the pathogenicity of each probable *LDLR* variant and the potential pathogenicity of each residue (**Figure** **8**). Figure 8A,B are similar to Figure 4 and 5D, respectively. However, for the optimization the scale has been increased, being the maximum value 7.

Finally, we measured the performance of the optimized model in each LDLr domain (**Table** **3**). Except for the specificity on the O‐linked domain, the accuracy of the model is >90%, concluding that it can predict variant pathogenicity with high accuracy in any domain. Statistics of each software are shown in Table S2 (Supporting Information).

**Table 3:** Accuracy (Sn, Sp and predicted mutations) of the optimized model for each domain and the whole protein.

Domain	Statistic	Numb	%	Domain	Statistic	Numb	%
Signal sequence	Sn	4	100	EGF‐C	Sn	34	92
	Sp	3	100		Sp	3	100
LBD	Sn	292	98	O‐Linked	Sn	0	0
	Sp	19	95		Sp	6	100
EGF‐A	Sn	20	95	Transm	Sn	1	100
	Sp	1	100		Sp	2	100
EGF‐B	Sn	37	100	Cytosolic	Sn	12	86
	Sp	2	100		Sp	2	100
B‐prop	Sn	191	95	**Total**	Sn	586	96
	Sp	21	100		Sp	60	98

Variants annotated on ClinVar database were used to test the accuracy of the software. Sn = Sensitivity; Sp = Specificity; Numb = Number of variants correctly predicted; % = Percentage of variants correctly predicted; LBD = Ligand Binding Domain; EGF‐A = Epidermal Growth Factor A domain; EGF‐B = Epidermal Growth Factor B domain; B‐prop = β‐propeller; EGF‐C = Epidermal Growth Factor C domain; Transm = Transmembrane domain.

### 3.4 Experimental Validation of the Model

To validate the model, we obtained the predicted pathogenicity scores of 93 *LDLR* variants classified as Benign/Likely Benign /Pathogenic/Likely Pathogenic by the ClinVar expert panel. These 93 variants were independently classified by the experts at ClinVar after the development of OptiMo‐LDLr. We used a threshold of 3.5 to distinguish between pathogenic and benign variants.

A comparison was then made between the model's predicted pathogenicity values and the classifications assigned by the ClinVar expert panel. Out of the 93 *LDLR* variants, OptiMo‐LDLr correctly predicts 95.7% of the variants. Notably, it correctly classifies 100% of the benign variants (5/5) and 95.4% of the pathogenic variants (84/88). Table S3 (Supporting Information) shows that only the values of 5 variants differed from the assigned classification by the ClinVar expert panel. This outcome supports the accuracy of the model in predicting the pathogenicity of these variants.

### 3.5 The Optimized Potential Pathogenicity Allows to Map LDLr Pathogenic Hotspots

**Figure 9:**
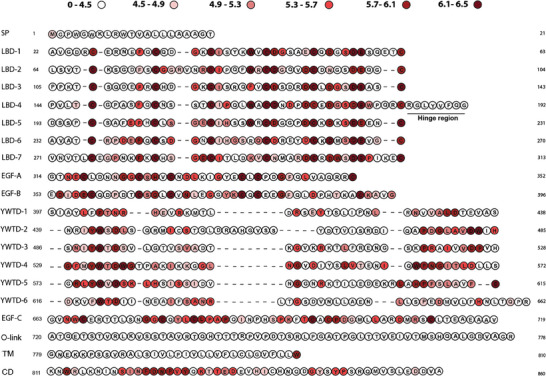
Potential pathogenicity map of LDLr residues. Each colour represents the potential pathogenicity ranges indicated at the top of the figure. LDLr domains and subdomains are indicated at the left of the image (SP: signal peptide; LBD: ligand binding domain; O‐link: O‐linked domain; TM: transmembrane domain; CD: cytosolic domain). Regarding the LBD domain, the seven LA repeats in which the domain is divided are indicated and the β‐propeller is divided into the six YWTD sequences. The alignment of LBD and β‐propeller domains has been performed following the one previously described.^[^ ^18^, ^19^ ^]^

Once the optimized potential pathogenicity of each residue was determined, these values were used to map the biological significance of each LDLr residue, identifying the calculated potential pathogenicity values with different colors (**Figure** **9**). This allowed to illustrate LDLr regions of biological importance, i.e., hot spots.

As shown in Figure 9, hot spot residues with high potential of being pathogenic when substituted appear across all domains except for the signal peptide, the O‐linked domain and the transmembrane domain. It is important to note that each 6 cysteines of the LBD are predicted as hot spot residues. On the other hand, the residues of the LBDs linker sequences are more permissive to be substituted presenting low pathogenicity potential. Regarding the β‐propeller, as shown in Figure 9, the identified hot spot residues are those responsible of maintaining the domain's conformation. Finally, the residues within NPxY domain show also high potential pathogenicity (Figure 9).

**Figure 10:**
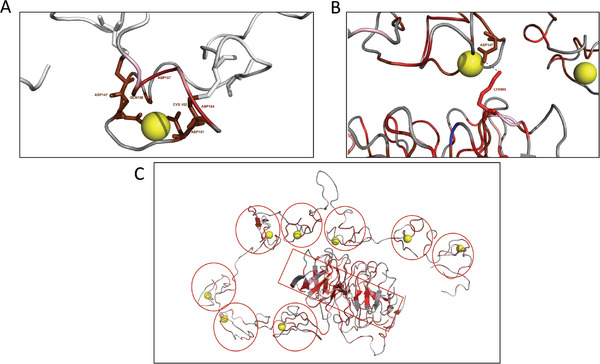
Potentially pathogenic clusters within the different LDLr domains. A) the hot spots detected in the LBD‐4 form a cluster that coordinates the calcium ion, and the cysteines maintain the subdomain's structure. B) Residues very distant in the primary structure that maintain the hinge allowing the closed conformation of the receptor at acidic pH. C) the 6 YWTD repeats arranged in 4 β‐sheets β‐propellers maintaining the architecture of the LDLr (red rectangle) and the LBD repetitions coordinating calcium to maintain the LDB subdomain's structure (red circles).

In addition, translating this information into 3D structure of the LDLr highlights potentially pathogenic clusters within the different domains, which might be related to specific biological function. Clearly, when analysing the hot spot residues beyond the primary structure, 3D mutational hotspots are revealed, grouped as clusters of amino acids performing different functions. In example, as shown in **Figure** **10**A, the hot spots detected in the LBD‐4 form a cluster that coordinates the calcium ion and the cysteines maintain the subdomain's structure. Very interestingly, residues that are very distant in the primary structure cluster into a 3D hot spot as shown by residues of the binding domain with those of the β‐propeller which constitute the hinge. This interaction maintains the closed conformation of the receptor at acidic pH to allow receptor's recycling function (Figure 10B). As shown in Figure 10B, the two hot spot residues allow the interaction of both domains at acidic pH. Finally, as shown in Figure 10C, the 6 YWTD repeats arranged in 4 β‐sheets β‐propellers have a specific folding pattern that brings neighbouring modules into close proximity and may have important consequences for the architecture of the LDLr, constitute a 3D hot spot cluster, which indicates the importance of the residues involved in maintaining this structural domain of the LDLr. Also, in Figure 10C it is shown the importance of calcium coordination in the LBD, each LBD repetition forms a cluster that maintaining the subdomain's structure.

## 4 Discussion

This study focuses on enhancing *LDLR* pathogenic variant prediction by integrating existing software that predicts the effects of mutations on LDLR. The development of OptiMo‐LDLr model significantly improves the predictive accuracy for *LDLR* variant pathogenicity and also allows to map hot spot residues of the LDLr causing FH. OptiMo‐LDLr model shows higher reliability than the individual ones and as the result of optimizing each software contribution, this model provides a higher predictive power than each software when tested individually both in pathogenic and benign *LDLR* variants.

OptiMo‐LDLr has the highest specificity and sensitivity values (specificity of 96.71% and a sensitivity of 98.36%). As expected, the algorithm resulting from software integration provided here is the most balanced model with an AUROC value only surpassed by VARITY. This is because the used algorithm improves the model for a certain threshold instead of for any possible one. By doing so, the obtained model is the most accurate one for those specific conditions. However, the fact that the non‐optimized model surpasses most of the predictive software suggests that the combination of them is a good predictive method.

Another good feature of OptiMo‐LDLr is that high predictive accuracy is maintained throughout the entire protein, as shown in Table 3. Despite the limited number of annotated variants in ClinVar for certain LDLr domains, the model exhibits a performance exceeding 90% accuracy in the majority of these domains. On the other hand, the obtained potential pathogenicity values and the ClinVar annotated information about LDLr agree, ensuring the usefulness of the approach. The accuracy of OptiMo‐LDLr was assessed through an independent evaluation of predicted pathogenicity for 93 *LDLR* variants, which had been classified by the expert panel at ClinVar. Notably, these variants were deliberately excluded from the training and validation datasets. The results demonstrated a substantial concurrence between OptiMo‐LDLr predictions, and the classifications made by the expert panel, affirming the robustness and reliability of the model.

The LDLr is recognized for its high cysteine content, and the formation of disulphide bonds between pairs of cysteines plays a pivotal role in ensuring the correct folding of its ten major functional modules.^[^ ^19^, ^20^ ^]^ According to data sourced from the ClinVar database, there have been identified missense mutations in 60 out of the 63 cysteines of the LDLr (with 95.2% of these variants being classified as pathogenic). Additionally, OptiMo‐LDLr identifies 60 out of those 63 cysteines as highly pathogenic, underscoring the significance of these residues in maintaining the protein's functionality. Interestingly, the ClinVar database also shows a high number of mutations occurring in Trp and Asp residues, following the pattern seen with cysteines. Among them, ClinVar shows that in 65% of Trp, pathogenic mutations have been found, and in 47% of the Asp. Moreover, the prediction of the OptiMo‐LDLr identifies 25 out of the 35 Asp residues and 12 out of the 20 Trp residues as highly pathogenic. On the other hand, only 8 of the 40 Asp in which no pathogenic mutation has been found, present a high value of potential pathogenicity. In the case of Trp, 3 of the 7 residues have a high pathogenicity value. These findings provide robust evidence underscoring the critical significance of these specific residues in ensuring the proper functioning of the LDLr.

Using OptiMo‐LDLr, we can infer detailed functional information about the hot spot residues within each LDLr domain. As an example, we will briefly examine the ligand‐binding domain, β‐propeller, O‐glycosylated, and cytosolic domains.

The ligand‐binding domain of the LDLr is composed of 7 A‐type repeating sequences of ≈40 amino acids each (LA repeats), represented as LBD‐1, LBD‐2, etc. Each LBD contains 6 cysteine residues that form three disulphide bridges (C1‐3, C2‐5, C4‐6), supporting the domain structure.^[^ ^18^, ^19^ ^]^ Interestingly, our analysis identified all of these cysteines as high‐potential pathogenicity residues on the resulting hot spot map, with 35 out of 42 classified with the maximum pathogenicity. Moreover, the Ca^+2^ coordination region between the fifth and sixth cysteines of each LBD, necessary for correct folding, was also identified as important on the potential pathogenicity map.^[^ ^21^ ^]^

The linker sequence between the LA repeats, consisting of 4 amino acids preceding the first cysteine of the LA repeat, was predicted to have low potential pathogenicity, consistent with experimentally described data.^[^ ^22^ ^]^ Additionally, a high potential pathogenicity value was predicted for the threonine typically located at position 4 of these linker sequences, potentially due to O‐glycosylation, which plays a key role in the proper structure of LBD and its interaction with ApoB‐100.^[^ ^23^, ^24^, ^25^ ^]^

In our examination of the functional implications of LR motifs in the LDL receptor's ligand‐binding domain, we specifically focused on exon 4. Exon 4 contains the three central LR motifs (LR3, LR4, and LR5), which have been considered to contain 3–4 times more mutations compared to other regions of the *LDLR* gene. To date, there are 61 variants affecting exon 4 of the *LDLR*. To gain further insights, we calculated the mutation index normalized by exon length, enabling a fair comparison of mutation frequencies in different exons, considering their varying lengths and the overall mutation rate across all exons. Our analysis revealed a mutation index normalized by exon length value of 1.51 for exon 4, significantly higher than the average mutation index of *LDLR* exons, which is 0.32 ± 0.15. Interestingly, the mutation frequency in exon 4 of the *LDLR* was calculated to be 4.8 times higher than that of the remaining exons in agreement with previously reported data. This finding indicates a relatively higher concentration of mutations within exon 4 compared to the average mutation rate observed across all exons, highlighting its potential functional significance. Notably, all 18 cysteine residues within Exon 4 have been identified with pathogenic mutations. This reinforces the notion that these cysteine residues serve as pivotal hot spots within the LDLr.

Moreover, a high mutation index normalized by exon length has been observed for exon 9, which at 1.7 and with a mutation frequency of 6.6 surpasses that of exon 4. It is noteworthy that although these exons exhibit a higher number of mutations, a significant portion of them occurs at the same amino acid position, thereby reinforcing the concept of hot spots. These findings underscore the importance of exons 4 and 9 in the mutational landscape of the *LDLR* and support their potential functional relevance.

Exon 9 codifies YWTD‐2 and YWTD‐3 two of the 6 blades that scaffold the β‐propeller. The β‐propeller is a structural domain formed by 6 YWTD repeats arranged in 4 β‐sheets. This structure is essential for the release of LDL in low pH environments, such as endosome, which allows LDLr recycling. As indicated by OptiMo‐LDLr, residues sustaining this conformation, such as YWTD repeats and glutamic acid, exhibit elevated potential pathogenicity, underscoring their physiological significance.^[^ ^26^ ^]^ The results provided by OptiMo‐LDLr identifies the Tyr and Trp residues of YWTD‐^[^ ^2^, ^3^, ^4^, ^5^ ^]^ as high‐potential pathogenicity residues on the resulting hot spot map. It has been shown that the side chains of hydrophobic amino acids on YWTD‐2 and YWTD‐3 contact with the linker that connects the β‐propeller to the C‐terminal EGF‐like module, thus positioning this module in contact with the second and third blades of the β‐propeller.^[^ ^27^ ^]^ High number of mutations within the YWTD region of the LDLr are already annotated in ClinVar and alter crucial conserved scaffolding residues of the β‐propeller. The results obtained from OptiMo‐LDLr indicate that the YWTD region, particularly YWTD‐2 and YWTD‐3, represents critical hot spot essential for maintaining the structural integrity and functional activity of the LDLr β‐propeller domain.

As for O‐glycosylated domain, very low potential pathogenicity residues have been predicted. This is consistent with the data obtained in ClinVar, where most variants located within this domain are classified as benign.^[^ ^6^ ^]^ It has been proposed that the function of this domain is to stabilize the receptor in the membrane.^[^ ^28^ ^]^ This could be the reason of its low potential pathogenicity values.

Finally, areas of high potential pathogenicity have been found in the cytosolic domain, especially at the beginning of it (FDNPVY). These residues correspond to the NPxY domain, which has been described as relevant on the internalization of LDLr,^[^ ^29^ ^]^ confirming their biological importance. Moreover, translating the identified hot spots in the primary sequence into 3D structure of the LDLr delineates 3D hotspot regions on the level of amino acid positions, 3D protein structure and the biological functions.

Considering the similarity between the optimized potential pathogenicity values and the known information about LDLr functional sites, the LDLr hot spot mapping presented here could be used as a tool for identification of pathogenic residues.

A potential avenue for future research involves extending the OptiMo‐LDLr model to predict pathogenicity for other genes linked to FH, such as *APOB* and *PCSK9*. However, this expansion faces a challenge due to the limited availability of well‐characterized variant databases for these proteins. A more extensive dataset is crucial for unlocking the full potential of the model in predicting pathogenicity for *APOB* and *PCSK9*, emphasizing the need for continued efforts in comprehensive variant characterization and data accumulation.

In conclusion, the integration of predictive software tools in OptiMo‐LDLr demonstrates notable reclassification power to pathogenicity, surpassing the individual use of isolated software. This is evident from the elevated specificity and sensitivity values attained by the optimized model. Moreover, the ability of OptiMo‐LDLr to effectively identify pathogenic hot spots across the entire protein reinforces its superior reclassification potential. OptiMo‐LDLr is a more robust and precise prediction approach, making it a valuable tool for advancing genetic diagnoses and providing comprehensive insights into the pathogenicity of *LDLR* variants associated with Familial Hypercholesterolemia.

### 4.1 Study Limitations

SFIP‐MutID was excluded from this work as its method for predicting pathogenicity resulted not compatible with the rest of the software. In fact, SFIP‐MutID estimates the pathogenicity by analyzing different structural characteristics without weighting their importance. Therefore, any adaptation of their score to the one used in this work could distort its meaning.

As it has been concluded by the study of the standard deviation among normalized pathogenicity values, the divergence of predictions between software varies according to the regions of LDLr. The prediction accuracy improves with increased agreement among the software in the combination. Besides, the properties of the variants used to optimize the combination of software cause the predictions to be more reliable in some domains than in others.

### 4.2 Clinical perspectives

Despite being one of the most prevalent genetic disorders, FH is frequently underdiagnosed. Developing novel in silico tools for predicting pathogenicity holds promise for enhancing the diagnosis and management of the disease. This study demonstrates that, notwithstanding the variations in predictions among different software, a synergistic integration of their results strengthens the predictive capacity, thereby facilitating more accurate genetic diagnoses. OptiMo‐LDLr not only enables precise classification of pathogenic variants but also harbors significant clinical implications, providing a user‐friendly interface for the scientific community to explore and apply in both research and clinical settings.

## Conflict of Interest

The authors declare no conflict of interest.

## Supporting information

Supporting Information

## Data Availability

The data that support the findings of this study are available from the corresponding author upon reasonable request.
