# Supplementary material for: OptiMo‐LDLr: An Integrated In Silico Model with Enhanced Predictive Power for LDL Receptor Variants, Unraveling Hot Spot Pathogenic Residues
Source: Adv Sci (Weinh). 2024 Jan 23;11(13):2305177. doi: 10.1002/advs.202305177 (PMC10987110; doi:10.1002/advs.202305177)
Supplement: Supplementary file 1 — Supporting Information [file ADVS-11-2305177-s001.pdf]

ADVANCED SCIENCE
Open Access

Supporting Information

for *Adv. Sci.*, DOI 10.1002/advs.202305177

OptiMo-LDLr: An Integrated In Silico Model with Enhanced Predictive Power for LDL Receptor Variants, Unraveling Hot Spot Pathogenic Residues

*Asier Larrea-Sebal, Iñaki Sasiain, Shifa Jebari-Benslaiman, Unai Galicia-Garcia, Kepa B. Uribe, Asier Benito-Vicente, Irene Gracia-Rubio, Harbil Bediaga-Bañeres, Sonia Arrasate, Ana Cenarro, Fernando Civeira, Humberto González-Díaz and Cesar Martín**

Supporting information

Full Title: OptiMo-LDLr: An Integrated In Silico Model with Enhanced Predictive Power for LDL Receptor Variants, Unraveling Hot Spot Pathogenic Residues

Authors´names: A. Larrea-Sebal[a,b,c], I. Sasiain[b], S. Jebari-Benslaiman[a,b], U. Galicia-Garcia[a,b], K. B. Uribe [b], A. Benito-Vicente[a,b], I. Gracia-Rubio[d], H. Bediaga-Bañeres[e], S. Arrasate[f], A. Cenarro[d], F. Civeir [d], H. González-Díaz[a,g], C. Martín[a,b]

Author affiliations: [a] Biofisika Institute (UPV/EHU, CSIC), Barrio Sarriena s/n., 48940 Leioa, Bizkaia, Spain. [b] Department of Biochemistry and Molecular Biology, Universidad del País Vasco UPV/EHU, 48940 Leioa, Bizkaia, Spain. [c] Fundación Biofisika Bizkaia, Barrio Sarriena s/n., 48940 Leioa, Bizkaia, Spain. [d] Lipid Unit, Hospital Universitario Miguel Servet, IIS Aragon, CIBERCV, Universidad de Zaragoza, Spain. [e] Department of Physical Chemistry, University of Basque Country UPV/EHU, 48940, Leioa, Spain. [f] Department of Organic and Chemistry, University of the Basque Country UPV/EHU, Leioa, Spain. [g] Ikerbasque, Basque Foundation for Science, 48013 Bilbao, Bizkaia, Spain.

Corresponding author:
Dr. César Martín
Dpt. Biochemistry and Molecular Biology, Science and Technology School.
University of the Basque Country (UPV/EHU)
Barrio Sarriena s/n 48080 Bilbao, Spain
Email: cesar.martin@ehu.eus Phone: +(34) 94 601 8052

Supplementary Methods

Excel Solver Evolutionary Algorithm (ESEA)

Excel Solver acts on a group of cells that are directly or indirectly related to the objective function. Modifying the value of those variables, it is able to optimize the target cell value, increasing or decreasing it. The objective of this step was to optimize the number of correctly predicted mutations modifying the adjustable values e_s coefficients. Excel Solver has three resolution methods: GRG Nonlinear, Simplex LP and Evolutionary. The Excel Solver Evolutionary Algorithm method was used, since it was the one that best fits this problem. ESEA applies the principles of evolution found in nature, giving random values at first, mutating or changing them and picking the best offspring to start again. That randomness makes the algorithm nondeterministic, so each new run can yield a different solution, and it can be an infinite process, since it has no concept of an optimal solution. However, it also helps avoiding local optimum values, as the algorithm uses different populations. Several trials with different starting values were performed to bypass non-optimum local maximum values, and the one with the best score was chosen. Overall, ESEA is the most fitting option for problems in which it is difficult to test the optimality of the results.

Table S1: Predictions of analyzed software and new models about ClinVar database.

ClinVar Code	Variant	T/V	CV	SIFT	Poly	Mut	MLb	REVEL	VERITY	Opti
NM_000527.5(LDLR):c.3G>A (p.Met1Ile)	MET1ILE	T	P	P	P	B	B	P	P	P
NM_000527.5(LDLR):c.1A>G (p.Met1Val)	MET1VAL	T	P	P	B	P	B	P	P	P
NM_000527.5(LDLR):c.28T>C (p.Trp10Arg)	TRP10ARG	T	P	P	P	B	P	P	B	P
NM_000527.5(LDLR):c.44T>A (p.Leu15His)	LEU15HIS	V	P	P	P	P	B	P	B	P
NM_000527.5(LDLR):c.53C>T (p.Ala18Val)	ALA18VAL	T	B	B	P	P	P	P	P	P
NM_000527.5(LDLR):c.58G>A (p.Gly20Arg)	GLY20ARG	T	B	P	P	P	P	B	P	P
NM_000527.5(LDLR):c.59G>A (p.Gly20Glu)	GLY20GLU	T	B	P	P	P	P	P	P	P
NM_000527.5(LDLR):c.79_81delinsCGT (p.Cys27Arg)	CYS27ARG	T	P	P	P	P	P	P	P	P
NM_000527.5(LDLR):c.95T>G (p.Phe32Cys)	PHE32CYS	T	P	P	P	P	P	P	P	P
NM_000527.5(LDLR):c.100T>G (p.Cys34Gly)	CYS34GLY	T	P	P	P	P	P	P	P	P
NM_000527.5(LDLR):c.101G>C (p.Cys34Ser)	CYS34SER	T	P	P	P	P	P	P	P	P
NM_000527.5(LDLR):c.102C>G (p.Cys34Trp)	CYS34TRP	T	P	P	P	B	P	P	P	P
NM_000527.5(LDLR):c.108C>A (p.Asp36Glu)	ASP36GLU	V	P	B	P	B	P	P	B	P
NM_000527.5(LDLR):c.115T>C (p.Cys39Arg)	CYS39ARG	T	P	P	P	P	P	P	P	P
NM_000527.5(LDLR):c.116G>T (p.Cys39Phe)	CYS39PHE	T	P	P	P	P	P	P	P	P
NM_000527.5(LDLR):c.136T>G (p.Cys46Gly)	CYS46GLY	T	P	P	P	P	P	P	P	P
NM_000527.5(LDLR):c.137G>C (p.Cys46Ser)	CYS46SER	T	P	P	P	P	P	P	P	P
NM_000527.5(LDLR):c.137G>A (p.Cys46Tyr)	CYS46TYR	T	P	P	P	P	P	P	P	P
NM_000527.5(LDLR):c.139G>C (p.Asp47His)	ASP47HIS	T	P	P	P	P	P	P	P	P
NM_000527.5(LDLR):c.155G>A (p.Cys52Tyr)	CYS52TYR	T	P	P	P	P	P	P	P	P
NM_000527.5(LDLR):c.166T>C (p.Ser56Pro)	SER56PRO	T	P	P	P	P	P	P	P	P
NM_000527.5(LDLR):c.173A>G (p.Glu58Gly)	GLU58GLY	T	P	P	P	P	P	P	P	P
NM_000527.5(LDLR):c.172G>A (p.Glu58Lys)	GLU58LYS	T	P	P	P	P	P	P	P	P
NM_000527.5(LDLR):c.187T>C (p.Cys63Arg)	CYS63ARG	T	P	P	P	P	P	P	P	P
NM_000527.5(LDLR):c.188G>T (p.Cys63Phe)	CYS63PHE	T	P	P	P	P	P	P	P	P
NM_000527.5(LDLR):c.223T>A (p.Cys75Ser)	CYS75SER	T	P	P	P	P	P	P	P	P
NM_000527.5(LDLR):c.225T>G (p.Cys75Trp)	CYS75TRP	V	P	P	P	B	P	P	P	P
NM_000527.5(LDLR):c.224G>A (p.Cys75Tyr)	CYS75TYR	T	P	P	P	P	P	P	P	P
NM_000527.5(LDLR):c.241C>A (p.Arg81Ser)	ARG81SER	T	P	P	P	P	B	P	P	P

NM_000527.5(LDLR):c.244T>G (p.Cys82Gly)	CYS82GLY	T	P	P	P	P	P	P	P	P
NM_000527.5(LDLR):c.245G>T (p.Cys82Phe)	CYS82PHE	T	P	P	P	P	P	P	P	P
NM_000527.5(LDLR):c.245G>A (p.Cys82Tyr)	CYS82TYR	T	P	P	P	P	P	P	P	P
NM_000527.5(LDLR):c.248T>C (p.Ile83Thr)	ILE83THR	T	P	P	P	P	P	P	P	P
NM_000527.5(LDLR):c.250C>T (p.Pro84Ser)	PRO84SER	T	B	B	B	P	B	B	P	P
NM_000527.5(LDLR):c.251C>G (p.Pro84Arg)	PRO84ARG	V	P	B	P	B	P	P	P	P
NM_000527.5(LDLR):c.251C>T (p.Pro84Leu)	PRO84LEU	V	P	P	P	P	P	P	P	P
NM_000527.5(LDLR):c.259T>C (p.Trp87Arg)	TRP87ARG	T	P	P	P	P	P	P	P	P
NM_000527.5(LDLR):c.259T>G (p.Trp87Gly)	TRP87GLY	T	P	P	P	P	P	P	P	P
NM_000527.5(LDLR):c.262A>G (p.Arg88Gly)	ARG88GLY	V	P	B	P	P	P	P	P	P
NM_000527.5(LDLR):c.263G>A (p.Arg88Lys)	ARG88LYS	T	P	B	B	P	P	B	B	B
NM_000527.5(LDLR):c.265T>C (p.Cys89Arg)	CYS89ARG	T	P	P	P	P	P	P	P	P
NM_000527.5(LDLR):c.265T>G (p.Cys89Gly)	CYS89GLY	T	P	P	P	P	P	P	P	P
NM_000527.5(LDLR):c.267C>G (p.Cys89Trp)	CYS89TRP	T	P	P	P	B	P	P	P	P
NM_000527.5(LDLR):c.266G>A (p.Cys89Tyr)	CYS89TYR	T	P	P	P	P	P	P	P	P
NM_000527.5(LDLR):c.269A>C (p.Asp90Ala)	ASP90ALA	T	P	P	P	P	P	P	P	P
NM_000527.5(LDLR):c.270T>A (p.Asp90Glu)	ASP90GLU	V	P	P	P	B	P	P	P	P
NM_000527.5(LDLR):c.269A>G (p.Asp90Gly)	ASP90GLY	T	P	P	P	P	P	P	P	P
NM_000527.5(LDLR):c.268G>T (p.Asp90Tyr)	ASP90TYR	T	P	P	P	P	P	P	P	P
NM_000527.5(LDLR):c.280G>T (p.Asp94Tyr)	ASP94TYR	T	P	P	P	P	P	P	P	P
NM_000527.5(LDLR):c.283T>C (p.Cys95Arg)	CYS95ARG	T	P	P	P	P	P	P	P	P
NM_000527.5(LDLR):c.284G>T (p.Cys95Phe)	CYS95PHE	T	P	P	P	P	P	P	P	P
NM_000527.5(LDLR):c.283T>A (p.Cys95Ser)	CYS95SER	T	P	P	P	P	P	P	P	P
NM_000527.5(LDLR):c.291C>G (p.Asn97Lys)	ASN97LYS	V	P	P	P	P	P	P	P	P
NM_000527.5(LDLR):c.300C>G (p.Asp100Glu)	ASP100GLU	T	P	P	P	B	P	P	P	P
NM_000527.5(LDLR):c.299A>G (p.Asp100Gly)	ASP100GLY	V	P	P	P	P	P	P	P	P
NM_000527.5(LDLR):c.302A>G (p.Glu101Gly)	GLU101GLY	T	P	P	P	P	P	P	P	P
NM_000527.5(LDLR):c.301G>A (p.Glu101Lys)	GLU101LYS	T	P	P	P	P	P	P	P	P
NM_000527.5(LDLR):c.310T>C (p.Cys104Arg)	CYS104ARG	V	P	P	P	P	P	P	P	P
NM_000527.5(LDLR):c.310T>G (p.Cys104Gly)	CYS104GLY	T	P	P	P	P	P	P	P	P
NM_000527.5(LDLR):c.311G>C (p.Cys104Ser)	CYS104SER	T	P	P	P	P	P	P	P	P
NM_000527.5(LDLR):c.312T>G (p.Cys104Trp)	CYS104TRP	T	P	P	P	B	P	P	P	P
NM_000527.5(LDLR):c.311G>A (p.Cys104Tyr)	CYS104TYR	V	P	P	P	P	P	P	P	P
NM_000527.5(LDLR):c.324_325delinsTC (p.Cys109Arg)	CYS109ARG	V	P	P	P	P	P	P	P	P
NM_000527.5(LDLR):c.325T>G (p.Cys109Gly)	CYS109GLY	T	P	P	P	P	P	P	P	P
NM_000527.5(LDLR):c.326G>T (p.Cys109Phe)	CYS109PHE	T	P	P	P	P	P	P	P	P
NM_000527.5(LDLR):c.326G>C (p.Cys109Ser)	CYS109SER	T	P	P	P	P	P	P	P	P
NM_000527.5(LDLR):c.326G>A (p.Cys109Tyr)	CYS109TYR	T	P	P	P	P	P	P	P	P
NM_000527.5(LDLR):c.343C>T (p.Arg115Cys)	ARG115CYS	T	P	B	P	P	P	P	P	P
NM_000527.5(LDLR):c.346T>C (p.Cys116Arg)	CYS116ARG	V	P	P	P	P	P	P	P	P
NM_000527.5(LDLR):c.347G>T (p.Cys116Phe)	CYS116PHE	T	P	P	P	P	P	P	P	P

NM_000527.5(LDLR):c.349C>T (p.His117Tyr)	HIS117TYR	T	B	P	P	P	P	B	P	P
NM_000527.5(LDLR):c.355G>A (p.Gly119Arg)	GLY119ARG	V	P	B	P	P	P	P	P	P
NM_000527.5(LDLR):c.361T>C (p.Cys121Arg)	CYS121ARG	T	P	P	P	P	P	P	P	P
NM_000527.5(LDLR):c.361T>G (p.Cys121Gly)	CYS121GLY	T	P	P	P	P	P	P	P	P
NM_000527.5(LDLR):c.362G>T (p.Cys121Phe)	CYS121PHE	T	P	P	P	P	P	P	P	P
NM_000527.5(LDLR):c.361T>A (p.Cys121Ser)	CYS121SER	T	P	P	P	P	P	P	P	P
NM_000527.5(LDLR):c.363C>G (p.Cys121Trp)	CYS121TRP	T	P	P	P	B	P	P	P	P
NM_000527.5(LDLR):c.362G>A (p.Cys121Tyr)	CYS121TYR	T	P	P	P	P	P	P	P	P
NM_000527.5(LDLR):c.364A>T (p.Ile122Phe)	ILE122PHE	T	P	P	P	P	B	P	P	P
NM_000527.5(LDLR):c.367T>A (p.Ser123Thr)	SER123THR	V	B	P	P	P	P	B	P	P
NM_000527.5(LDLR):c.370C>G (p.Arg124Gly)	ARG124GLY	T	B	P	B	P	P	B	P	P
NM_000527.5(LDLR):c.373C>A (p.Gln125Lys)	GLN125LYS	T	B	P	P	P	P	B	P	P
NM_000527.5(LDLR):c.376T>C (p.Phe126Leu)	PHE126LEU	T	P	P	P	P	P	P	P	P
NM_000527.5(LDLR):c.377T>C (p.Phe126Ser)	PHE126SER	V	P	P	P	P	P	P	P	P
NM_000527.5(LDLR):c.377T>A (p.Phe126Tyr)	PHE126TYR	V	P	B	P	P	P	P	P	P
NM_000527.5(LDLR):c.380T>A (p.Val127Asp)	VAL127ASP	V	P	P	P	P	P	P	P	P
NM_000527.5(LDLR):c.380T>G (p.Val127Gly)	VAL127GLY	T	P	P	B	P	B	P	P	P
NM_000527.5(LDLR):c.382T>C (p.Cys128Arg)	CYS128ARG	T	P	P	P	P	P	P	P	P
NM_000527.5(LDLR):c.382T>G (p.Cys128Gly)	CYS128GLY	T	P	P	P	P	P	P	P	P
NM_000527.5(LDLR):c.383G>T (p.Cys128Phe)	CYS128PHE	V	P	P	P	P	P	P	P	P
NM_000527.5(LDLR):c.383G>C (p.Cys128Ser)	CYS128SER	V	P	P	P	P	P	P	P	P
NM_000527.5(LDLR):c.383G>A (p.Cys128Tyr)	CYS128TYR	T	P	P	P	P	P	P	P	P
NM_000527.5(LDLR):c.386A>G (p.Asp129Gly)	ASP129GLY	T	P	P	P	P	P	P	P	P
NM_000527.5(LDLR):c.391G>C (p.Asp131His)	ASP131HIS	T	P	P	P	P	P	P	P	P
NM_000527.5(LDLR):c.397G>A (p.Asp133Asn)	ASP133ASN	V	P	B	P	P	P	P	P	P
NM_000527.5(LDLR):c.400T>C (p.Cys134Arg)	CYS134ARG	V	P	P	P	P	P	P	P	P
NM_000527.5(LDLR):c.400T>G (p.Cys134Gly)	CYS134GLY	V	P	P	P	P	P	P	P	P
NM_000527.5(LDLR):c.401G>T (p.Cys134Phe)	CYS134PHE	T	P	P	P	P	P	P	P	P
NM_000527.5(LDLR):c.401G>C (p.Cys134Ser)	CYS134SER	V	P	P	P	P	P	P	P	P
NM_000527.5(LDLR):c.402C>G (p.Cys134Trp)	CYS134TRP	T	P	P	P	B	P	P	P	P
NM_000527.5(LDLR):c.401G>A (p.Cys134Tyr)	CYS134TYR	T	P	P	P	P	P	P	P	P

NM_000527.5(LDLR):c.407A>T (p.Asp136Val)	ASP136VAL	T	P	P	P	P	P	P	P	P
NM_000527.5(LDLR):c.409G>T (p.Gly137Cys)	GLY137CYS	V	P	P	P	P	P	P	P	P
NM_000527.5(LDLR):c.415G>A (p.Asp139Asn)	ASP139ASN	V	P	P	P	P	P	P	P	P
NM_000527.5(LDLR):c.417C>A (p.Asp139Glu)	ASP139GLU	V	P	P	P	B	P	P	P	P
NM_000527.5(LDLR):c.416A>G (p.Asp139Gly)	ASP139GLY	T	P	P	P	P	P	P	P	P
NM_000527.5(LDLR):c.415G>C (p.Asp139His)	ASP139HIS	V	P	P	P	P	P	P	P	P
NM_000527.5(LDLR):c.416A>T (p.Asp139Val)	ASP139VAL	T	P	P	P	P	P	P	P	P
NM_000527.5(LDLR):c.420G>T (p.Glu140Asp)	GLU140ASP	T	P	P	P	B	P	P	P	P
NM_000527.5(LDLR):c.419A>G (p.Glu140Gly)	GLU140GLY	T	P	P	P	P	P	P	P	P
NM_000527.5(LDLR):c.418G>A (p.Glu140Lys)	GLU140LYS	T	P	P	P	P	P	P	P	P
NM_000527.5(LDLR):c.427T>C (p.Cys143Arg)	CYS143ARG	V	P	P	P	P	P	P	P	P
NM_000527.5(LDLR):c.427T>G (p.Cys143Gly)	CYS143GLY	T	P	P	P	P	P	P	P	P
NM_000527.5(LDLR):c.428G>T (p.Cys143Phe)	CYS143PHE	T	P	P	P	P	P	P	P	P
NM_000527.5(LDLR):c.427T>A (p.Cys143Ser)	CYS143SER	V	P	P	P	P	P	P	P	P
NM_000527.5(LDLR):c.428G>A (p.Cys143Tyr)	CYS143TYR	V	P	P	P	P	P	P	P	P
NM_000527.5(LDLR):c.442T>C (p.Cys148Arg)	CYS148ARG	T	P	P	P	P	P	P	P	P
NM_000527.5(LDLR):c.443G>C (p.Cys148Ser)	CYS148SER	T	P	P	P	P	P	P	P	P
NM_000527.5(LDLR):c.444T>G (p.Cys148Trp)	CYS148TRP	T	P	P	P	B	P	P	P	P
NM_000527.5(LDLR):c.443G>A (p.Cys148Tyr)	CYS148TYR	T	P	P	P	P	P	P	P	P
NM_000527.5(LDLR):c.445G>C (p.Gly149Arg)	GLY149ARG	T	P	P	P	B	P	B	B	P
NM_000527.5(LDLR):c.451G>A (p.Ala151Thr)	ALA151THR	T	B	P	P	P	P	B	P	P
NM_000527.5(LDLR):c.458T>G (p.Phe153Cys)	PHE153CYS	T	P	P	P	P	P	P	P	P
NM_000527.5(LDLR):c.457T>G (p.Phe153Val)	PHE153VAL	T	P	P	P	P	P	P	P	P
NM_000527.5(LDLR):c.463T>C (p.Cys155Arg)	CYS155ARG	T	P	P	P	P	P	P	P	P
NM_000527.5(LDLR):c.463T>G (p.Cys155Gly)	CYS155GLY	V	P	P	P	P	P	P	P	P
NM_000527.5(LDLR):c.464G>T (p.Cys155Phe)	CYS155PHE	V	P	P	P	P	P	P	P	P
NM_000527.5(LDLR):c.463T>A (p.Cys155Ser)	CYS155SER	T	P	P	P	P	P	P	P	P
NM_000527.5(LDLR):c.464G>A (p.Cys155Tyr)	CYS155TYR	T	P	P	P	P	P	P	P	P
NM_000527.5(LDLR):c.470G>A (p.Ser157Asn)	SER157ASN	T	P	B	B	B	B	B	B	B
NM_000527.5(LDLR):c.473C>G (p.Ser158Cys)	SER158CYS	T	P	B	P	B	B	B	P	P
NM_000527.5(LDLR):c.476C>T (p.Thr159Ile)	THR159ILE	T	B	P	P	P	P	B	P	P

NM_000527.5(LDLR):c.478T>C (p.Cys160Arg)	CYS160ARG	T	P	P	P	P	P	P	P	P
NM_000527.5(LDLR):c.478T>G (p.Cys160Gly)	CYS160GLY	T	P	P	P	P	P	P	P	P
NM_000527.5(LDLR):c.479G>T (p.Cys160Phe)	CYS160PHE	T	P	P	P	P	P	P	P	P
NM_000527.5(LDLR):c.478T>A (p.Cys160Ser)	CYS160SER	T	P	P	P	P	P	P	P	P
NM_000527.5(LDLR):c.482T>A (p.Ile161Asn)	ILE161ASN	V	P	P	P	P	B	P	P	P
NM_000527.5(LDLR):c.482T>C (p.Ile161Thr)	ILE161THR	T	P	P	P	P	P	P	P	P
NM_000527.5(LDLR):c.485C>T (p.Pro162Leu)	PRO162LEU	T	P	P	P	P	P	P	P	P
NM_000527.5(LDLR):c.493T>G (p.Trp165Gly)	TRP165GLY	T	P	P	P	P	P	P	P	P
NM_000527.5(LDLR):c.499T>C (p.Cys167Arg)	CYS167ARG	T	P	P	P	P	P	P	P	P
NM_000527.5(LDLR):c.501C>G (p.Cys167Trp)	CYS167TRP	T	P	P	P	B	P	P	P	P
NM_000527.5(LDLR):c.500G>A (p.Cys167Tyr)	CYS167TYR	T	P	P	P	P	P	P	P	P
NM_000527.5(LDLR):c.503A>C (p.Asp168Ala)	ASP168ALA	T	P	P	P	P	P	P	P	P
NM_000527.5(LDLR):c.502G>A (p.Asp168Asn)	ASP168ASN	T	P	P	P	P	P	P	P	P
NM_000527.5(LDLR):c.504C>A (p.Asp168Glu)	ASP168GLU	T	P	P	P	B	P	P	P	P
NM_000527.5(LDLR):c.503A>G (p.Asp168Gly)	ASP168GLY	T	P	P	P	P	P	P	P	P
NM_000527.5(LDLR):c.502G>C (p.Asp168His)	ASP168HIS	T	P	P	P	P	P	P	P	P
NM_000527.5(LDLR):c.502G>T (p.Asp168Tyr)	ASP168TYR	T	P	P	P	P	P	P	P	P
NM_000527.5(LDLR):c.506A>T (p.Asn169Ile)	ASN169ILE	T	P	P	P	P	P	P	P	P
NM_000527.5(LDLR):c.514G>A (p.Asp172Asn)	ASP172ASN	T	P	P	P	P	P	P	P	P
NM_000527.5(LDLR):c.516C>G (p.Asp172Glu)	ASP172GLU	T	P	P	P	B	P	P	P	P
NM_000527.5(LDLR):c.515A>G (p.Asp172Gly)	ASP172GLY	T	P	P	P	P	P	P	P	P
NM_000527.5(LDLR):c.514G>C (p.Asp172His)	ASP172HIS	T	P	P	P	P	P	P	P	P
NM_000527.5(LDLR):c.514G>T (p.Asp172Tyr)	ASP172TYR	T	P	P	P	P	P	P	P	P
NM_000527.5(LDLR):c.517T>C (p.Cys173Arg)	CYS173ARG	T	P	P	P	P	P	P	P	P
NM_000527.5(LDLR):c.517T>G (p.Cys173Gly)	CYS173GLY	T	P	P	P	P	P	P	P	P
NM_000527.5(LDLR):c.518G>C (p.Cys173Ser)	CYS173SER	T	P	P	P	P	P	P	P	P
NM_000527.5(LDLR):c.519C>G (p.Cys173Trp)	CYS173TRP	T	P	P	P	B	P	P	P	P
NM_000527.5(LDLR):c.518G>A (p.Cys173Tyr)	CYS173TYR	V	P	P	P	P	P	P	P	P
NM_000527.5(LDLR):c.523G>A (p.Asp175Asn)	ASP175ASN	V	P	P	P	P	P	P	P	P
NM_000527.5(LDLR):c.523G>T (p.Asp175Tyr)	ASP175TYR	T	P	P	P	P	P	P	P	P
NM_000527.5(LDLR):c.527G>T (p.Gly176Val)	GLY176VAL	T	P	P	P	P	P	P	P	P

NM_000527.5(LDLR):c.530C>T (p.Ser177Leu)	SER177LEU	T	P	P	P	P	P	P	P	P
NM_000527.5(LDLR):c.529T>C (p.Ser177Pro)	SER177PRO	T	P	P	P	P	P	P	P	P
NM_000527.5(LDLR):c.532G>A (p.Asp178Asn)	ASP178ASN	T	P	P	P	P	P	P	P	P
NM_000527.5(LDLR):c.534T>G (p.Asp178Glu)	ASP178GLU	T	P	P	P	B	P	P	P	P
NM_000527.5(LDLR):c.533A>G (p.Asp178Gly)	ASP178GLY	T	P	P	P	P	P	P	P	P
NM_000527.5(LDLR):c.532G>C (p.Asp178His)	ASP178HIS	T	P	P	P	P	P	P	P	P
NM_000527.5(LDLR):c.532G>T (p.Asp178Tyr)	ASP178TYR	T	P	P	P	P	P	P	P	P
NM_000527.5(LDLR):c.533A>T (p.Asp178Val)	ASP178VAL	T	P	P	P	P	P	P	P	P
NM_000527.5(LDLR):c.536A>G (p.Glu179Gly)	GLU179GLY	T	P	P	P	P	P	P	P	P
NM_000527.5(LDLR):c.535G>A (p.Glu179Lys)	GLU179LYS	T	P	P	P	P	P	P	P	P
NM_000527.5(LDLR):c.542C>T (p.Pro181Leu)	PRO181LEU	T	P	B	B	P	P	P	B	P
NM_000527.5(LDLR):c.548G>A (p.Arg183His)	ARG183HIS	T	B	P	P	P	P	P	P	P
NM_000527.5(LDLR):c.550T>C (p.Cys184Arg)	CYS184ARG	T	P	P	P	P	P	P	P	P
NM_000527.5(LDLR):c.552T>G (p.Cys184Trp)	CYS184TRP	V	P	P	P	B	P	P	P	P
NM_000527.5(LDLR):c.551G>A (p.Cys184Tyr)	CYS184TYR	T	P	P	P	P	P	P	P	P
NM_000527.5(LDLR):c.565G>A (p.Val189Met)	VAL189MET	T	B	P	B	P	P	B	P	P
NM_000527.5(LDLR):c.584G>A (p.Ser195Asn)	SER195ASN	V	B	P	P	P	P	P	P	P
NM_000527.5(LDLR):c.586C>A (p.Pro196Thr)	PRO196THR	V	P	B	P	P	P	P	P	P
NM_000527.5(LDLR):c.589T>C (p.Cys197Arg)	CYS197ARG	V	P	P	P	P	P	P	P	P
NM_000527.5(LDLR):c.589T>G (p.Cys197Gly)	CYS197GLY	T	P	P	P	P	P	P	P	P
NM_000527.5(LDLR):c.590G>T (p.Cys197Phe)	CYS197PHE	V	P	P	P	P	P	P	P	P
NM_000527.5(LDLR):c.591C>G (p.Cys197Trp)	CYS197TRP	T	P	P	P	B	P	P	P	P
NM_000527.5(LDLR):c.590G>A (p.Cys197Tyr)	CYS197TYR	T	P	P	P	P	P	P	P	P
NM_000527.5(LDLR):c.600C>A (p.Phe200Leu)	PHE200LEU	T	B	P	P	P	B	P	P	P
NM_000527.5(LDLR):c.601G>A (p.Glu201Lys)	GLU201LYS	V	P	P	P	P	P	P	P	P
NM_000527.5(LDLR):c.605T>C (p.Phe202Ser)	PHE202SER	T	P	P	P	P	P	P	P	P
NM_000527.5(LDLR):c.610T>G (p.Cys204Gly)	CYS204GLY	T	P	P	P	P	P	P	P	P
NM_000527.5(LDLR):c.611G>T (p.Cys204Phe)	CYS204PHE	V	P	P	P	P	P	P	P	P
NM_000527.5(LDLR):c.611G>C (p.Cys204Ser)	CYS204SER	T	P	P	P	P	P	P	P	P
NM_000527.5(LDLR):c.611G>A (p.Cys204Tyr)	CYS204TYR	V	P	P	P	P	P	P	P	P
NM_000527.5(LDLR):c.613C>G (p.Leu205Val)	LEU205VAL	T	P	B	B	B	P	B	B	B

NM_000527.5(LDLR):c.622G>A (p.Glu208Lys)	GLU208LYS	T	P	B	P	P	P	P	P	P
NM_000527.5(LDLR):c.625T>G (p.Cys209Gly)	CYS209GLY	T	P	P	P	P	P	P	P	P
NM_000527.5(LDLR):c.626G>A (p.Cys209Tyr)	CYS209TYR	T	P	P	P	P	P	P	P	P
NM_000527.5(LDLR):c.629T>A (p.Ile210Asn)	ILE210ASN	T	P	P	P	P	P	P	P	P
NM_000527.5(LDLR):c.628A>C (p.Ile210Leu)	ILE210LEU	V	P	P	B	P	B	P	B	B
NM_000527.5(LDLR):c.629T>C (p.Ile210Thr)	ILE210THR	T	P	P	P	P	P	P	P	P
NM_000527.5(LDLR):c.641G>C (p.Trp214Ser)	TRP214SER	T	P	P	P	P	P	P	P	P
NM_000527.5(LDLR):c.644G>A (p.Arg215His)	ARG215HIS	V	P	B	B	P	P	P	B	B
NM_000527.5(LDLR):c.643C>A (p.Arg215Ser)	ARG215SER	T	P	B	P	P	B	P	P	P
NM_000527.5(LDLR):c.646T>C (p.Cys216Arg)	CYS216ARG	T	P	P	P	P	P	P	P	P
NM_000527.5(LDLR):c.647G>T (p.Cys216Phe)	CYS216PHE	V	P	P	P	P	P	P	P	P
NM_000527.5(LDLR):c.647G>A (p.Cys216Tyr)	CYS216TYR	V	P	P	P	P	P	P	P	P
NM_000527.5(LDLR):c.661G>A (p.Asp221Asn)	ASP221ASN	V	P	P	P	P	P	P	P	P
NM_000527.5(LDLR):c.662A>G (p.Asp221Gly)	ASP221GLY	T	P	P	P	P	P	P	P	P
NM_000527.5(LDLR):c.661G>T (p.Asp221Tyr)	ASP221TYR	T	P	P	P	P	P	P	P	P
NM_000527.5(LDLR):c.662A>T (p.Asp221Val)	ASP221VAL	T	P	P	P	P	P	P	P	P
NM_000527.5(LDLR):c.664T>G (p.Cys222Gly)	CYS222GLY	T	P	P	P	P	P	P	P	P
NM_000527.5(LDLR):c.665G>T (p.Cys222Phe)	CYS222PHE	T	P	P	P	P	P	P	P	P
NM_000527.5(LDLR):c.666C>G (p.Cys222Trp)	CYS222TRP	T	P	P	P	B	P	P	P	P
NM_000527.5(LDLR):c.665G>A (p.Cys222Tyr)	CYS222TYR	T	P	P	P	P	P	P	P	P
NM_000527.5(LDLR):c.669G>C (p.Lys223Asn)	LYS223ASN	T	P	B	P	P	B	B	P	P
NM_000527.5(LDLR):c.671A>C (p.Asp224Ala)	ASP224ALA	V	P	P	P	P	P	P	P	P
NM_000527.5(LDLR):c.670G>A (p.Asp224Asn)	ASP224ASN	T	P	P	P	P	P	P	P	P
NM_000527.5(LDLR):c.671A>G (p.Asp224Gly)	ASP224GLY	T	P	P	P	P	P	P	P	P
NM_000527.5(LDLR):c.671A>T (p.Asp224Val)	ASP224VAL	V	P	P	P	P	P	P	P	P
NM_000527.5(LDLR):c.677C>G (p.Ser226Cys)	SER226CYS	T	P	P	P	P	P	P	P	P
NM_000527.5(LDLR):c.676T>C (p.Ser226Pro)	SER226PRO	T	P	P	P	P	P	P	P	P
NM_000527.5(LDLR):c.681C>A (p.Asp227Glu)	ASP227GLU	V	P	P	P	B	P	P	P	P
NM_000527.5(LDLR):c.680A>T (p.Asp227Val)	ASP227VAL	T	P	P	P	P	P	P	P	P
NM_000527.5(LDLR):c.683A>C (p.Glu228Ala)	GLU228ALA	V	P	P	P	P	P	P	P	P
NM_000527.5(LDLR):c.684G>T (p.Glu228Asp)	GLU228ASP	T	P	B	P	B	P	P	P	P

NM_000527.5(LDLR):c.682G>C (p.Glu228Gln)	GLU228GLN	T	P	P	P	P	P	P	P	P
NM_000527.5(LDLR):c.683A>G (p.Glu228Gly)	GLU228GLY	T	P	P	P	P	P	P	P	P
NM_000527.5(LDLR):c.682G>A (p.Glu228Lys)	GLU228LYS	T	P	P	P	P	P	P	P	P
NM_000527.5(LDLR):c.691T>C (p.Cys231Arg)	CYS231ARG	V	P	P	P	P	P	P	P	P
NM_000527.5(LDLR):c.691T>G (p.Cys231Gly)	CYS231GLY	T	P	P	P	P	P	P	P	P
NM_000527.5(LDLR):c.693C>G (p.Cys231Trp)	CYS231TRP	T	P	P	P	B	P	P	P	P
NM_000527.5(LDLR):c.692G>A (p.Cys231Tyr)	CYS231TYR	V	P	P	P	P	P	P	P	P
NM_000527.5(LDLR):c.706T>C (p.Cys236Arg)	CYS236ARG	T	P	P	P	P	P	P	P	P
NM_000527.5(LDLR):c.706T>G (p.Cys236Gly)	CYS236GLY	V	P	P	P	P	P	P	P	P
NM_000527.5(LDLR):c.722T>G (p.Phe241Cys)	PHE241CYS	T	P	P	P	P	P	P	P	P
NM_000527.5(LDLR):c.722T>C (p.Phe241Ser)	PHE241SER	V	P	P	P	P	P	P	P	P
NM_000527.5(LDLR):c.737G>T (p.Gly246Val)	GLY246VAL	T	P	P	P	P	P	P	P	P
NM_000527.5(LDLR):c.742T>G (p.Cys248Gly)	CYS248GLY	V	P	P	P	P	P	P	P	P
NM_000527.5(LDLR):c.743G>T (p.Cys248Phe)	CYS248PHE	T	P	P	P	P	P	P	P	P
NM_000527.5(LDLR):c.743G>C (p.Cys248Ser)	CYS248SER	T	P	P	P	P	P	P	P	P
NM_000527.5(LDLR):c.743G>A (p.Cys248Tyr)	CYS248TYR	T	P	P	P	P	P	P	P	P
NM_000527.5(LDLR):c.746T>C (p.Ile249Thr)	ILE249THR	T	P	P	P	P	P	P	P	P
NM_000527.5(LDLR):c.758G>C (p.Arg253Pro)	ARG253PRO	T	P	B	P	P	P	P	P	P
NM_000527.5(LDLR):c.762G>T (p.Gln254His)	GLN254HIS	V	P	P	P	B	P	P	P	P
NM_000527.5(LDLR):c.761A>C (p.Gln254Pro)	GLN254PRO	T	P	P	P	P	P	P	P	P
NM_000527.5(LDLR):c.762_763inv (p.Cys255Arg)	CYS255ARG	T	P	P	P	P	P	P	P	P
NM_000527.5(LDLR):c.763T>G (p.Cys255Gly)	CYS255GLY	V	P	P	P	P	P	P	P	P
NM_000527.5(LDLR):c.763T>A (p.Cys255Ser)	CYS255SER	V	P	P	P	P	P	P	P	P
NM_000527.5(LDLR):c.764G>A (p.Cys255Tyr)	CYS255TYR	T	P	P	P	P	P	P	P	P
NM_000527.5(LDLR):c.767A>G (p.Asp256Gly)	ASP256GLY	T	P	P	P	P	P	P	P	P
NM_000527.5(LDLR):c.770G>T (p.Arg257Leu)	ARG257LEU	V	B	P	B	P	P	B	P	P
NM_000527.5(LDLR):c.770G>C (p.Arg257Pro)	ARG257PRO	T	P	B	P	B	B	P	P	P
NM_000527.5(LDLR):c.772G>A (p.Glu258Lys)	GLU258LYS	V	B	P	P	B	P	B	P	P
NM_000527.5(LDLR):c.782G>T (p.Cys261Phe)	CYS261PHE	V	P	P	P	P	P	P	P	P
NM_000527.5(LDLR):c.782G>A (p.Cys261Tyr)	CYS261TYR	T	P	P	P	P	P	P	P	P
NM_000527.5(LDLR):c.790A>G (p.Met264Val)	MET264VAL	T	B	P	B	B	B	P	P	P

NM_000527.5(LDLR):c.796G>A (p.Asp266Asn)	ASP266ASN	T	P	P	P	P	P	P	P	P
NM_000527.5(LDLR):c.798T>A (p.Asp266Glu)	ASP266GLU	T	P	P	P	B	P	P	P	P
NM_000527.5(LDLR):c.796G>T (p.Asp266Tyr)	ASP266TYR	T	P	P	P	P	P	P	P	P
NM_000527.5(LDLR):c.797A>T (p.Asp266Val)	ASP266VAL	T	P	P	P	P	P	P	P	P
NM_000527.5(LDLR):c.801A>T (p.Glu267Asp)	GLU267ASP	V	P	P	P	B	P	P	P	P
NM_000527.5(LDLR):c.806G>A (p.Gly269Asp)	GLY269ASP	T	B	P	P	B	B	B	B	P
NM_000527.5(LDLR):c.805G>A (p.Gly269Ser)	GLY269SER	T	P	B	P	P	P	P	P	P
NM_000527.5(LDLR):c.808T>C (p.Cys270Arg)	CYS270ARG	T	P	P	P	P	P	P	P	P
NM_000527.5(LDLR):c.808T>A (p.Cys270Ser)	CYS270SER	T	P	P	P	P	P	P	P	P
NM_000527.5(LDLR):c.810C>G (p.Cys270Trp)	CYS270TRP	T	P	P	P	B	P	P	P	P
NM_000527.5(LDLR):c.809G>A (p.Cys270Tyr)	CYS270TYR	T	P	P	P	P	P	P	P	P
NM_000527.5(LDLR):c.820A>T (p.Thr274Ser)	THR274SER	V	B	P	P	B	P	B	P	P
NM_000527.5(LDLR):c.826T>G (p.Cys276Gly)	CYS276GLY	T	P	P	P	P	P	P	P	P
NM_000527.5(LDLR):c.828C>G (p.Cys276Trp)	CYS276TRP	V	P	P	P	B	P	P	P	P
NM_000527.5(LDLR):c.827G>A (p.Cys276Tyr)	CYS276TYR	T	P	P	P	P	P	P	P	P
NM_000527.5(LDLR):c.850T>C (p.Cys284Arg)	CYS284ARG	V	P	P	P	P	P	P	P	P
NM_000527.5(LDLR):c.850T>G (p.Cys284Gly)	CYS284GLY	V	P	P	P	P	P	P	P	P
NM_000527.5(LDLR):c.851G>T (p.Cys284Phe)	CYS284PHE	T	P	P	P	P	P	P	P	P
NM_000527.5(LDLR):c.855C>A (p.His285Gln)	HIS285GLN	T	B	P	P	P	B	B	P	P
NM_000527.5(LDLR):c.859G>T (p.Gly287Cys)	GLY287CYS	T	P	P	P	P	P	P	P	P
NM_000527.5(LDLR):c.862G>A (p.Glu288Lys)	GLU288LYS	T	P	B	P	P	P	P	P	P
NM_000527.5(LDLR):c.865T>G (p.Cys289Gly)	CYS289GLY	T	P	P	P	P	P	P	P	P
NM_000527.5(LDLR):c.869T>G (p.Ile290Ser)	ILE290SER	T	P	P	P	P	P	P	P	P
NM_000527.5(LDLR):c.880A>G (p.Lys294Glu)	LYS294GLU	V	P	B	P	P	P	P	P	P
NM_000527.5(LDLR):c.886T>C (p.Cys296Arg)	CYS296ARG	T	P	P	P	P	P	P	P	P
NM_000527.5(LDLR):c.887G>C (p.Cys296Ser)	CYS296SER	T	P	P	P	P	P	P	P	P
NM_000527.5(LDLR):c.887G>A (p.Cys296Tyr)	CYS296TYR	T	P	P	P	P	P	P	P	P
NM_000527.5(LDLR):c.895G>A (p.Ala299Thr)	ALA299THR	T	P	B	B	B	B	P	B	B
NM_000527.5(LDLR):c.902A>C (p.Asp301Ala)	ASP301ALA	T	P	P	P	P	P	P	P	P
NM_000527.5(LDLR):c.902A>G (p.Asp301Gly)	ASP301GLY	T	P	P	P	P	P	P	P	P
NM_000527.5(LDLR):c.901G>T (p.Asp301Tyr)	ASP301TYR	T	P	P	P	P	P	P	P	P

NM_000527.5(LDLR):c.904T>C (p.Cys302Arg)	CYS302ARG	T	P	P	P	P	P	P	P	P
NM_000527.5(LDLR):c.905G>T (p.Cys302Phe)	CYS302PHE	T	P	P	P	P	P	P	P	P
NM_000527.5(LDLR):c.906C>G (p.Cys302Trp)	CYS302TRP	T	P	P	P	B	P	P	P	P
NM_000527.5(LDLR):c.910G>A (p.Asp304Asn)	ASP304ASN	T	P	P	P	P	P	P	P	P
NM_000527.5(LDLR):c.912C>G (p.Asp304Glu)	ASP304GLU	V	P	P	P	B	P	P	P	P
NM_000527.5(LDLR):c.910G>C (p.Asp304His)	ASP304HIS	T	P	P	P	P	P	P	P	P
NM_000527.5(LDLR):c.910G>T (p.Asp304Tyr)	ASP304TYR	T	P	P	P	P	P	P	P	P
NM_000527.5(LDLR):c.911A>T (p.Asp304Val)	ASP304VAL	V	P	P	P	P	P	P	P	P
NM_000527.5(LDLR):c.915G>T (p.Trp305Cys)	TRP305CYS	T	P	P	P	B	P	P	P	P
NM_000527.5(LDLR):c.917C>T (p.Ser306Leu)	SER306LEU	T	P	P	P	P	P	P	P	P
NM_000527.5(LDLR):c.921T>G (p.Asp307Glu)	ASP307GLU	T	P	P	P	B	P	P	P	P
NM_000527.5(LDLR):c.920A>G (p.Asp307Gly)	ASP307GLY	T	P	P	P	P	P	P	P	P
NM_000527.5(LDLR):c.919G>C (p.Asp307His)	ASP307HIS	T	P	P	P	P	P	P	P	P
NM_000527.5(LDLR):c.924A>T (p.Glu308Asp)	GLU308ASP	T	P	P	P	B	P	P	P	P
NM_000527.5(LDLR):c.922G>A (p.Glu308Lys)	GLU308LYS	T	P	P	P	P	P	P	P	P
NM_000527.5(LDLR):c.926C>A (p.Pro309His)	PRO309HIS	T	P	P	P	P	P	P	P	P
NM_000527.5(LDLR):c.929T>C (p.Ile310Thr)	ILE310THR	V	B	P	B	B	B	B	P	P
NM_000527.5(LDLR):c.932A>C (p.Lys311Thr)	LYS311THR	T	P	P	P	P	P	P	P	P
NM_000527.5(LDLR):c.937T>G (p.Cys313Gly)	CYS313GLY	T	P	P	P	P	P	P	P	P
NM_000527.5(LDLR):c.938G>A (p.Cys313Tyr)	CYS313TYR	V	P	P	P	P	P	P	P	P
NM_000527.5(LDLR):c.947A>G (p.Asn316Ser)	ASN316SER	T	P	P	P	P	B	P	P	P
NM_000527.5(LDLR):c.947A>C (p.Asn316Thr)	ASN316THR	T	P	P	P	P	P	P	P	P
NM_000527.5(LDLR):c.952T>C (p.Cys318Arg)	CYS318ARG	T	P	P	P	P	P	P	P	P
NM_000527.5(LDLR):c.953G>T (p.Cys318Phe)	CYS318PHE	T	P	P	P	P	P	P	P	P
NM_000527.5(LDLR):c.953G>A (p.Cys318Tyr)	CYS318TYR	T	P	P	P	P	P	P	P	P
NM_000527.5(LDLR):c.965A>T (p.Asn322Ile)	ASN322ILE	T	P	P	P	P	P	P	P	P
NM_000527.5(LDLR):c.967G>T (p.Gly323Cys)	GLY323CYS	V	P	P	P	P	P	P	P	P
NM_000527.5(LDLR):c.970G>A (p.Gly324Ser)	GLY324SER	T	B	B	B	B	B	B	B	B
NM_000527.5(LDLR):c.973T>C (p.Cys325Arg)	CYS325ARG	T	P	P	P	P	P	P	P	P
NM_000527.5(LDLR):c.974G>T (p.Cys325Phe)	CYS325PHE	T	P	P	P	P	P	P	P	P
NM_000527.5(LDLR):c.977C>G (p.Ser326Cys)	SER326CYS	T	P	P	P	P	P	P	P	P

NM_000527.5(LDLR):c.977C>T (p.Ser326Phe)	SER326PHE	T	P	P	P	P	P	P	P	P
NM_000527.5(LDLR):c.981C>A (p.His327Gln)	HIS327GLN	T	P	B	P	B	P	P	P	P
NM_000527.5(LDLR):c.985T>C (p.Cys329Arg)	CYS329ARG	T	P	P	P	P	P	P	P	P
NM_000527.5(LDLR):c.985T>G (p.Cys329Gly)	CYS329GLY	V	P	P	P	P	P	P	P	P
NM_000527.5(LDLR):c.986G>T (p.Cys329Phe)	CYS329PHE	T	P	P	P	P	P	P	P	P
NM_000527.5(LDLR):c.987C>G (p.Cys329Trp)	CYS329TRP	T	P	P	P	B	P	P	P	P
NM_000527.5(LDLR):c.990T>A (p.Asn330Lys)	ASN330LYS	T	B	P	P	P	P	P	P	P
NM_000527.5(LDLR):c.1004G>A (p.Gly335Asp)	GLY335ASP	T	P	P	P	P	P	P	P	P
NM_000527.5(LDLR):c.1004G>T (p.Gly335Val)	GLY335VAL	T	P	P	P	P	P	P	P	P
NM_000527.5(LDLR):c.1012T>C (p.Cys338Arg)	CYS338ARG	T	P	P	P	P	P	P	P	P
NM_000527.5(LDLR):c.1012T>G (p.Cys338Gly)	CYS338GLY	T	P	P	P	P	P	P	P	P
NM_000527.5(LDLR):c.1013G>T (p.Cys338Phe)	CYS338PHE	T	P	P	P	P	P	P	P	P
NM_000527.5(LDLR):c.1012T>A (p.Cys338Ser)	CYS338SER	T	P	P	P	P	P	P	P	P
NM_000527.5(LDLR):c.1014C>G (p.Cys338Trp)	CYS338TRP	V	P	P	P	B	P	P	P	P
NM_000527.5(LDLR):c.1013G>A (p.Cys338Tyr)	CYS338TYR	#N/D	P	P	P	P	P	P	P	P
NM_000527.5(LDLR):c.1016T>C (p.Leu339Pro)	LEU339PRO	T	P	B	P	P	P	P	P	P
NM_000527.5(LDLR):c.1019_1020delinsTG (p.Cys340Leu)	CYS340LEU	V	P	P	P	P	P	P	P	P
NM_000527.5(LDLR):c.1019G>T (p.Cys340Phe)	CYS340PHE	T	P	P	P	P	P	P	P	P
NM_000527.5(LDLR):c.1020C>G (p.Cys340Trp)	CYS340TRP	T	P	P	P	B	P	P	P	P
NM_000527.5(LDLR):c.1022C>G (p.Pro341Arg)	PRO341ARG	T	B	P	P	B	B	B	P	P
NM_000527.5(LDLR):c.1028G>A (p.Gly343Asp)	GLY343ASP	T	P	P	P	P	P	P	P	P
NM_000527.5(LDLR):c.1027G>T (p.Gly343Cys)	GLY343CYS	V	P	P	P	P	P	P	P	P
NM_000527.5(LDLR):c.1028G>T (p.Gly343Val)	GLY343VAL	T	P	P	P	P	P	P	P	P
NM_000527.5(LDLR):c.1049G>A (p.Arg350Gln)	ARG350GLN	T	P	B	P	B	B	B	B	B
NM_000527.5(LDLR):c.1054T>C (p.Cys352Arg)	CYS352ARG	T	P	P	P	P	P	P	P	P
NM_000527.5(LDLR):c.1055G>T (p.Cys352Phe)	CYS352PHE	T	P	P	P	P	P	P	P	P
NM_000527.5(LDLR):c.1054T>A (p.Cys352Ser)	CYS352SER	T	P	P	P	P	P	P	P	P
NM_000527.5(LDLR):c.1056C>G (p.Cys352Trp)	CYS352TRP	T	P	P	P	B	P	P	P	P
NM_000527.5(LDLR):c.1055G>A (p.Cys352Tyr)	CYS352TYR	T	P	P	P	P	P	P	P	P
NM_000527.5(LDLR):c.1061A>C (p.Asp354Ala)	ASP354ALA	T	P	P	P	P	P	P	P	P
NM_000527.5(LDLR):c.1061A>G (p.Asp354Gly)	ASP354GLY	V	P	P	P	P	P	P	P	P

NM_000527.5(LDLR):c.1061A>T (p.Asp354Val)	ASP354VAL	T	P	P	P	P	P	P	P	P
NM_000527.5(LDLR):c.1067A>C (p.Asp356Ala)	ASP356ALA	T	P	P	P	P	P	P	P	P
NM_000527.5(LDLR):c.1067A>T (p.Asp356Val)	ASP356VAL	V	P	P	P	P	P	P	P	P
NM_000527.5(LDLR):c.1070A>G (p.Glu357Gly)	GLU357GLY	T	P	P	P	P	P	P	P	P
NM_000527.5(LDLR):c.1072T>C (p.Cys358Arg)	CYS358ARG	T	P	P	P	P	P	P	P	P
NM_000527.5(LDLR):c.1073G>A (p.Cys358Tyr)	CYS358TYR	T	P	P	P	P	P	P	P	P
NM_000527.5(LDLR):c.1081C>T (p.Pro361Ser)	PRO361SER	V	P	B	P	P	P	P	P	P
NM_000527.5(LDLR):c.1091G>T (p.Cys364Phe)	CYS364PHE	T	P	P	P	P	P	P	P	P
NM_000527.5(LDLR):c.1091G>C (p.Cys364Ser)	CYS364SER	V	P	P	P	P	P	P	P	P
NM_000527.5(LDLR):c.1091G>A (p.Cys364Tyr)	CYS364TYR	T	P	P	P	P	P	P	P	P
NM_000527.5(LDLR):c.1097A>G (p.Gln366Arg)	GLN366ARG	V	P	P	P	P	P	P	P	P
NM_000527.5(LDLR):c.1097A>C (p.Gln366Pro)	GLN366PRO	T	P	P	P	P	P	P	P	P
NM_000527.5(LDLR):c.1102T>C (p.Cys368Arg)	CYS368ARG	T	P	P	P	P	P	P	P	P
NM_000527.5(LDLR):c.1102T>G (p.Cys368Gly)	CYS368GLY	T	P	P	P	P	P	P	P	P
NM_000527.5(LDLR):c.1103G>C (p.Cys368Ser)	CYS368SER	T	P	P	P	P	P	P	P	P
NM_000527.5(LDLR):c.1110C>G (p.Asn370Lys)	ASN370LYS	T	P	P	P	P	P	P	P	P
NM_000527.5(LDLR):c.1109A>C (p.Asn370Thr)	ASN370THR	T	P	P	P	P	P	P	P	P
NM_000527.5(LDLR):c.1118G>C (p.Gly373Ala)	GLY373ALA	T	P	P	P	P	P	P	P	P
NM_000527.5(LDLR):c.1118G>A (p.Gly373Asp)	GLY373ASP	V	P	P	P	P	P	P	P	P
NM_000527.5(LDLR):c.1117G>T (p.Gly373Cys)	GLY373CYS	V	P	P	P	P	P	P	P	P
NM_000527.5(LDLR):c.1118G>T (p.Gly373Val)	GLY373VAL	T	P	P	P	P	P	P	P	P
NM_000527.5(LDLR):c.1120_1121delinsTC (p.Gly374Ser)	GLY374SER	T	B	P	P	B	P	P	P	P
NM_000527.5(LDLR):c.1124A>G (p.Tyr375Cys)	TYR375CYS	T	P	P	P	P	P	P	P	P
NM_000527.5(LDLR):c.1124A>C (p.Tyr375Ser)	TYR375SER	T	P	P	P	P	P	P	P	P
NM_000527.5(LDLR):c.1129T>G (p.Cys377Gly)	CYS377GLY	T	P	P	P	P	P	P	P	P
NM_000527.5(LDLR):c.1130G>T (p.Cys377Phe)	CYS377PHE	T	P	P	P	P	P	P	P	P
NM_000527.5(LDLR):c.1130G>C (p.Cys377Ser)	CYS377SER	T	P	P	P	P	P	P	P	P
NM_000527.5(LDLR):c.1130G>A (p.Cys377Tyr)	CYS377TYR	T	P	P	P	P	P	P	P	P
NM_000527.5(LDLR):c.1135T>C (p.Cys379Arg)	CYS379ARG	T	P	P	P	P	P	P	P	P
NM_000527.5(LDLR):c.1135T>G (p.Cys379Gly)	CYS379GLY	V	P	P	P	P	P	P	P	P
NM_000527.5(LDLR):c.1136G>A (p.Cys379Tyr)	CYS379TYR	T	P	P	P	P	P	P	P	P

NM_000527.5(LDLR):c.1151A>C (p.Gln384Pro)	GLN384PRO	T	P	B	P	P	P	P	P	P
NM_000527.5(LDLR):c.1154T>G (p.Leu385Arg)	LEU385ARG	T	P	P	P	P	P	P	P	P
NM_000527.5(LDLR):c.1154T>C (p.Leu385Pro)	LEU385PRO	T	P	B	P	P	P	P	P	P
NM_000527.5(LDLR):c.1171G>A (p.Ala391Thr)	ALA391THR	T	B	P	P	B	P	P	P	P
NM_000527.5(LDLR):c.1174T>C (p.Cys392Arg)	CYS392ARG	T	P	P	P	P	P	P	P	P
NM_000527.5(LDLR):c.1177A>C (p.Lys393Gln)	LYS393GLN	T	P	P	P	P	P	P	P	P
NM_000527.5(LDLR):c.1186G>C (p.Gly396Arg)	GLY396ARG	V	P	P	P	B	P	P	P	P
NM_000527.5(LDLR):c.1187G>A (p.Gly396Asp)	GLY396ASP	T	P	B	B	P	P	P	P	B
NM_000527.5(LDLR):c.1189T>A (p.Ser397Thr)	SER397THR	V	B	P	P	P	P	B	P	P
NM_000527.5(LDLR):c.1196C>A (p.Ala399Asp)	ALA399ASP	T	P	P	P	P	P	P	P	P
NM_000527.5(LDLR):c.1195G>T (p.Ala399Ser)	ALA399SER	T	P	B	B	P	B	P	B	B
NM_000527.5(LDLR):c.1205T>C (p.Phe402Ser)	PHE402SER	V	P	P	B	P	P	P	P	P
NM_000527.5(LDLR):c.1207T>C (p.Phe403Leu)	PHE403LEU	V	P	P	P	P	B	P	P	P
NM_000527.5(LDLR):c.1211C>T (p.Thr404Ile)	THR404ILE	V	P	P	P	P	P	P	P	P
NM_000527.5(LDLR):c.1210A>T (p.Thr404Ser)	THR404SER	T	P	B	B	P	P	P	P	P
NM_000527.5(LDLR):c.1215C>G (p.Asn405Lys)	ASN405LYS	T	P	P	P	P	P	P	P	P
NM_000527.5(LDLR):c.1217G>A (p.Arg406Gln)	ARG406GLN	V	P	P	P	P	B	P	P	P
NM_000527.5(LDLR):c.1217G>C (p.Arg406Pro)	ARG406PRO	T	P	P	P	P	P	P	P	P
NM_000527.5(LDLR):c.1216C>T (p.Arg406Trp)	ARG406TRP	T	P	P	P	P	P	P	P	P
NM_000527.5(LDLR):c.1223A>C (p.Glu408Ala)	GLU408ALA	T	P	P	P	P	P	P	P	P
NM_000527.5(LDLR):c.1222G>A (p.Glu408Lys)	GLU408LYS	T	P	P	P	P	P	P	P	P
NM_000527.5(LDLR):c.1223A>T (p.Glu408Val)	GLU408VAL	T	P	P	B	P	P	P	P	P
NM_000527.5(LDLR):c.1228A>G (p.Arg410Gly)	ARG410GLY	T	P	P	P	P	P	P	P	P
NM_000527.5(LDLR):c.1231A>G (p.Lys411Glu)	LYS411GLU	T	P	P	P	P	P	P	P	P
NM_000527.5(LDLR):c.1236G>A (p.Met412Ile)	MET412ILE	T	B	P	P	P	P	B	P	P
NM_000527.5(LDLR):c.1235T>C (p.Met412Thr)	MET412THR	T	P	P	P	P	B	P	P	P
NM_000527.5(LDLR):c.1238C>T (p.Thr413Met)	THR413MET	T	P	P	P	P	B	P	B	P
NM_000527.5(LDLR):c.1241T>G (p.Leu414Arg)	LEU414ARG	T	P	P	P	P	P	P	P	P
NM_000527.5(LDLR):c.1244A>G (p.Asp415Gly)	ASP415GLY	V	B	P	P	B	B	B	P	P
NM_000527.5(LDLR):c.1243G>C (p.Asp415His)	ASP415HIS	T	P	B	P	P	P	P	P	P
NM_000527.5(LDLR):c.1247G>T (p.Arg416Leu)	ARG416LEU	T	P	P	P	P	B	P	P	P

NM_000527.5(LDLR):c.1247G>C (p.Arg416Pro)	ARG416PRO	T	P	P	P	P	P	P	P	P
NM_000527.5(LDLR):c.1246C>T (p.Arg416Trp)	ARG416TRP	T	P	P	P	P	B	P	P	P
NM_000527.5(LDLR):c.1252G>A (p.Glu418Lys)	GLU418LYS	T	P	P	P	P	P	P	P	P
NM_000527.5(LDLR):c.1255T>G (p.Tyr419Asp)	TYR419ASP	V	P	P	P	P	P	P	P	P
NM_000527.5(LDLR):c.1256A>G (p.Tyr419Cys)	TYR419CYS	T	P	P	P	P	P	P	P	P
NM_000527.5(LDLR):c.1261A>G (p.Ser421Gly)	SER421GLY	V	B	P	B	B	P	P	B	P
NM_000527.5(LDLR):c.1265T>C (p.Leu422Pro)	LEU422PRO	T	P	P	P	P	P	P	P	P
NM_000527.5(LDLR):c.1268T>C (p.Ile423Thr)	ILE423THR	T	P	P	P	P	P	P	P	P
NM_000527.5(LDLR):c.1285G>T (p.Val429Leu)	VAL429LEU	V	P	B	B	P	B	P	P	B
NM_000527.5(LDLR):c.1285G>A (p.Val429Met)	VAL429MET	T	P	P	P	P	B	P	B	P
NM_000527.5(LDLR):c.1291G>C (p.Ala431Pro)	ALA431PRO	T	P	P	P	P	P	P	P	P
NM_000527.5(LDLR):c.1291G>A (p.Ala431Thr)	ALA431THR	T	P	P	P	P	P	P	P	P
NM_000527.5(LDLR):c.1295T>C (p.Leu432Pro)	LEU432PRO	T	P	P	P	P	P	P	P	P
NM_000527.5(LDLR):c.1297G>C (p.Asp433His)	ASP433HIS	T	P	P	P	P	P	P	P	P
NM_000527.5(LDLR):c.1297G>T (p.Asp433Tyr)	ASP433TYR	V	P	P	P	P	P	P	P	P
NM_000527.5(LDLR):c.1315A>T (p.Asn439Tyr)	ASN439TYR	T	P	P	P	P	P	P	P	P
NM_000527.5(LDLR):c.1322T>A (p.Ile441Asn)	ILE441ASN	T	P	P	P	P	P	P	P	P
NM_000527.5(LDLR):c.1323C>G (p.Ile441Met)	ILE441MET	T	P	P	P	B	B	P	B	P
NM_000527.5(LDLR):c.1322T>C (p.Ile441Thr)	ILE441THR	T	P	P	P	P	P	P	P	P
NM_000527.5(LDLR):c.1324T>A (p.Tyr442Asn)	TYR442ASN	T	P	P	P	P	P	P	P	P
NM_000527.5(LDLR):c.1325A>G (p.Tyr442Cys)	TYR442CYS	T	P	P	P	P	P	P	P	P
NM_000527.5(LDLR):c.1327T>C (p.Trp443Arg)	TRP443ARG	T	P	P	P	P	P	P	P	P
NM_000527.5(LDLR):c.1329G>T (p.Trp443Cys)	TRP443CYS	V	P	P	P	B	P	P	P	P
NM_000527.5(LDLR):c.1331C>T (p.Ser444Phe)	SER444PHE	T	P	P	P	P	P	P	P	P
NM_000527.5(LDLR):c.1330T>C (p.Ser444Pro)	SER444PRO	T	P	B	P	P	P	P	P	P
NM_000527.5(LDLR):c.1340C>G (p.Ser447Cys)	SER447CYS	V	P	P	P	P	P	P	P	P
NM_000527.5(LDLR):c.1339T>C (p.Ser447Pro)	SER447PRO	T	P	B	P	P	P	P	P	P
NM_000527.5(LDLR):c.1342C>A (p.Gln448Lys)	GLN448LYS	T	P	B	B	P	P	P	P	P
NM_000527.5(LDLR):c.1351A>T (p.Ile451Phe)	ILE451PHE	V	P	P	P	P	P	P	P	P
NM_000527.5(LDLR):c.1352T>C (p.Ile451Thr)	ILE451THR	T	P	P	P	P	P	P	P	P
NM_000527.5(LDLR):c.1361C>A (p.Thr454Asn)	THR454ASN	T	P	P	P	B	P	B	P	P

NM_000527.5(LDLR):c.1367T>A (p.Leu456His)	LEU456HIS	T	P	P	P	P	P	P	P	P
NM_000527.5(LDLR):c.1376C>G (p.Ala459Gly)	ALA459GLY	T	B	P	P	B	P	B	P	P
NM_000527.5(LDLR):c.1400C>T (p.Thr467Ile)	THR467ILE	V	B	P	P	P	P	B	P	P
NM_000527.5(LDLR):c.1408A>G (p.Ser470Gly)	SER470GLY	T	P	B	B	B	B	B	B	B
NM_000527.5(LDLR):c.1411A>G (p.Arg471Gly)	ARG471GLY	T	B	P	P	P	P	B	P	P
NM_000527.5(LDLR):c.1412G>A (p.Arg471Lys)	ARG471LYS	T	P	B	B	B	B	B	B	B
NM_000527.5(LDLR):c.1417A>G (p.Ile473Val)	ILE473VAL	V	B	P	P	P	P	P	P	P
NM_000527.5(LDLR):c.1424C>T (p.Ala475Val)	ALA475VAL	T	P	B	P	P	P	P	B	P
NM_000527.5(LDLR):c.1427C>T (p.Pro476Leu)	PRO476LEU	V	P	P	P	P	P	P	P	P
NM_000527.5(LDLR):c.1426C>T (p.Pro476Ser)	PRO476SER	T	P	P	P	P	P	P	P	P
NM_000527.5(LDLR):c.1433G>A (p.Gly478Glu)	GLY478GLU	T	P	P	P	P	P	P	P	P
NM_000527.5(LDLR):c.1436T>A (p.Leu479Gln)	LEU479GLN	V	P	P	P	P	P	P	P	P
NM_000527.5(LDLR):c.1436T>C (p.Leu479Pro)	LEU479PRO	T	P	P	P	P	P	P	P	P
NM_000527.5(LDLR):c.1438G>C (p.Ala480Pro)	ALA480PRO	T	P	P	P	P	P	P	P	P
NM_000527.5(LDLR):c.1438G>A (p.Ala480Thr)	ALA480THR	T	P	P	P	P	P	P	P	P
NM_000527.5(LDLR):c.1439C>T (p.Ala480Val)	ALA480VAL	V	P	P	P	P	P	P	P	P
NM_000527.5(LDLR):c.1444G>A (p.Asp482Asn)	ASP482ASN	T	P	P	P	P	P	P	P	P
NM_000527.5(LDLR):c.1445A>G (p.Asp482Gly)	ASP482GLY	V	P	P	P	P	P	P	P	P
NM_000527.5(LDLR):c.1444G>C (p.Asp482His)	ASP482HIS	T	P	P	P	P	P	P	P	P
NM_000527.5(LDLR):c.1444G>T (p.Asp482Tyr)	ASP482TYR	V	P	P	P	P	P	P	P	P
NM_000527.5(LDLR):c.1447T>C (p.Trp483Arg)	TRP483ARG	T	P	P	P	P	P	P	P	P
NM_000527.5(LDLR):c.1448G>T (p.Trp483Leu)	TRP483LEU	T	P	P	P	P	P	P	P	P
NM_000527.5(LDLR):c.1454A>G (p.His485Arg)	HIS485ARG	T	P	B	P	P	P	P	P	P
NM_000527.5(LDLR):c.1456A>G (p.Ser486Gly)	SER486GLY	V	B	P	P	B	P	B	P	P
NM_000527.5(LDLR):c.1463T>A (p.Ile488Asn)	ILE488ASN	T	P	P	P	P	P	P	P	P
NM_000527.5(LDLR):c.1463T>G (p.Ile488Ser)	ILE488SER	T	P	P	P	P	P	P	P	P
NM_000527.5(LDLR):c.1466A>G (p.Tyr489Cys)	TYR489CYS	V	P	P	P	P	P	P	P	P
NM_000527.5(LDLR):c.1468T>C (p.Trp490Arg)	TRP490ARG	T	P	P	P	P	P	P	P	P
NM_000527.5(LDLR):c.1468T>G (p.Trp490Gly)	TRP490GLY	T	P	P	P	P	P	P	P	P
NM_000527.5(LDLR):c.1469G>T (p.Trp490Leu)	TRP490LEU	T	P	P	P	P	P	P	P	P
NM_000527.5(LDLR):c.1472C>A (p.Thr491Asn)	THR491ASN	T	P	P	P	P	P	P	P	P

NM_000527.5(LDLR):c.1475A>G (p.Asp492Gly)	ASP492GLY	V	P	P	P	P	P	P	P	P
NM_000527.5(LDLR):c.1487G>T (p.Gly496Val)	GLY496VAL	T	P	B	P	P	P	P	P	P
NM_000527.5(LDLR):c.1489A>C (p.Thr497Pro)	THR497PRO	T	P	P	P	P	P	P	P	P
NM_000527.5(LDLR):c.1492G>T (p.Val498Phe)	VAL498PHE	T	B	B	P	B	B	B	B	P
NM_000527.5(LDLR):c.1502C>A (p.Ala501Glu)	ALA501GLU	V	P	P	P	P	P	P	P	P
NM_000527.5(LDLR):c.1501G>A (p.Ala501Thr)	ALA501THR	T	P	P	P	P	P	P	P	P
NM_000527.5(LDLR):c.1502C>T (p.Ala501Val)	ALA501VAL	T	P	P	B	P	P	P	P	P
NM_000527.5(LDLR):c.1514G>A (p.Gly505Asp)	GLY505ASP	T	P	P	P	P	P	P	P	P
NM_000527.5(LDLR):c.1521G>C (p.Lys507Asn)	LYS507ASN	T	P	B	P	P	B	P	P	P
NM_000527.5(LDLR):c.1520A>C (p.Lys507Thr)	LYS507THR	T	P	B	P	P	P	P	P	P
NM_000527.5(LDLR):c.1525A>G (p.Lys509Glu)	LYS509GLU	#N/D	P	B	P	P	P	P	P	P
NM_000527.5(LDLR):c.1532T>C (p.Leu511Ser)	LEU511SER	T	P	P	P	P	P	P	P	P
NM_000527.5(LDLR):c.1538G>A (p.Arg513Lys)	ARG513LYS	T	B	P	P	P	P	B	P	P
NM_000527.5(LDLR):c.1555C>T (p.Pro519Ser)	PRO519SER	T	P	P	P	P	P	P	P	P
NM_000527.5(LDLR):c.1558A>G (p.Arg520Gly)	ARG520GLY	T	P	B	P	P	P	P	P	P
NM_000527.5(LDLR):c.1567G>A (p.Val523Met)	VAL523MET	V	P	P	P	P	B	P	P	P
NM_000527.5(LDLR):c.1571T>G (p.Val524Gly)	VAL524GLY	T	P	P	P	P	P	P	P	P
NM_000527.5(LDLR):c.1574A>T (p.Asp525Val)	ASP525VAL	T	P	P	P	P	P	P	P	P
NM_000527.5(LDLR):c.1577C>G (p.Pro526Arg)	PRO526ARG	T	P	P	P	P	P	P	P	P
NM_000527.5(LDLR):c.1576C>A (p.Pro526Thr)	PRO526THR	T	P	P	P	P	P	P	P	P
NM_000527.5(LDLR):c.1586G>A (p.Gly529Asp)	GLY529ASP	V	P	P	P	B	P	P	P	P
NM_000527.5(LDLR):c.1592T>G (p.Met531Arg)	MET531ARG	T	P	P	P	P	P	P	P	P
NM_000527.5(LDLR):c.1594T>A (p.Tyr532Asn)	TYR532ASN	T	P	P	P	P	P	P	P	P
NM_000527.5(LDLR):c.1597T>C (p.Trp533Arg)	TRP533ARG	T	P	P	P	P	P	P	P	P
NM_000527.5(LDLR):c.1601C>A (p.Thr534Asn)	THR534ASN	V	P	P	P	P	P	P	P	P
NM_000527.5(LDLR):c.1603G>A (p.Asp535Asn)	ASP535ASN	V	P	P	B	P	P	P	P	P
NM_000527.5(LDLR):c.1603G>T (p.Asp535Tyr)	ASP535TYR	V	P	P	P	P	P	P	P	P
NM_000527.5(LDLR):c.1606T>G (p.Trp536Gly)	TRP536GLY	T	P	P	P	P	P	P	P	P
NM_000527.5(LDLR):c.1612A>T (p.Thr538Ser)	THR538SER	T	B	P	P	P	P	B	P	P
NM_000527.5(LDLR):c.1618G>A (p.Ala540Thr)	ALA540THR	T	P	P	P	P	P	P	P	P
NM_000527.5(LDLR):c.1625T>G (p.Ile542Ser)	ILE542SER	V	P	P	P	P	P	P	P	P

NM_000527.5(LDLR):c.1633G>T (p.Gly545Trp)	GLY545TRP	T	P	P	P	P	P	P	P	P
NM_000527.5(LDLR):c.1636G>C (p.Gly546Arg)	GLY546ARG	V	P	P	B	P	P	P	P	P
NM_000527.5(LDLR):c.1637G>A (p.Gly546Asp)	GLY546ASP	V	P	P	P	P	P	P	P	P
NM_000527.5(LDLR):c.1637G>T (p.Gly546Val)	GLY546VAL	T	P	P	P	P	P	P	P	P
NM_000527.5(LDLR):c.1644T>G (p.Asn548Lys)	ASN548LYS	T	P	P	P	P	P	B	P	P
NM_000527.5(LDLR):c.1646G>A (p.Gly549Asp)	GLY549ASP	T	P	P	P	P	P	P	P	P
NM_000527.5(LDLR):c.1646G>T (p.Gly549Val)	GLY549VAL	T	P	P	P	P	P	P	P	P
NM_000527.5(LDLR):c.1664T>C (p.Leu555Pro)	LEU555PRO	T	P	P	P	P	P	P	P	P
NM_000527.5(LDLR):c.1678A>T (p.Ile560Phe)	ILE560PHE	T	P	P	P	P	P	P	P	P
NM_000527.5(LDLR):c.1686G>T (p.Trp562Cys)	TRP562CYS	T	P	P	P	B	P	P	P	P
NM_000527.5(LDLR):c.1687C>T (p.Pro563Ser)	PRO563SER	T	P	P	P	P	P	P	P	P
NM_000527.5(LDLR):c.1691A>G (p.Asn564Ser)	ASN564SER	T	P	P	P	P	P	P	P	P
NM_000527.5(LDLR):c.1694G>C (p.Gly565Ala)	GLY565ALA	T	P	P	P	P	P	P	P	P
NM_000527.5(LDLR):c.1694G>T (p.Gly565Val)	GLY565VAL	V	P	P	P	P	P	P	P	P
NM_000527.5(LDLR):c.1703T>C (p.Leu568Pro)	LEU568PRO	T	P	P	P	P	P	P	P	P
NM_000527.5(LDLR):c.1702C>G (p.Leu568Val)	LEU568VAL	T	P	B	P	P	P	P	P	P
NM_000527.5(LDLR):c.1705G>A (p.Asp569Asn)	ASP569ASN	T	P	P	P	P	P	P	P	P
NM_000527.5(LDLR):c.1705G>T (p.Asp569Tyr)	ASP569TYR	T	P	P	B	P	P	P	P	P
NM_000527.5(LDLR):c.1715G>A (p.Ser572Asn)	SER572ASN	T	B	P	P	P	P	P	P	P
NM_000527.5(LDLR):c.1727A>C (p.Tyr576Ser)	TYR576SER	V	P	P	P	P	P	P	P	P
NM_000527.5(LDLR):c.1729T>C (p.Trp577Arg)	TRP577ARG	T	P	P	P	P	P	P	P	P
NM_000527.5(LDLR):c.1729T>G (p.Trp577Gly)	TRP577GLY	V	P	P	P	P	P	P	P	P
NM_000527.5(LDLR):c.1730G>C (p.Trp577Ser)	TRP577SER	T	P	P	P	P	P	P	P	P
NM_000527.5(LDLR):c.1733T>C (p.Val578Ala)	VAL578ALA	V	P	B	B	P	P	P	P	P
NM_000527.5(LDLR):c.1736A>C (p.Asp579Ala)	ASP579ALA	T	P	P	P	P	P	P	P	P
NM_000527.5(LDLR):c.1735G>A (p.Asp579Asn)	ASP579ASN	T	P	P	P	P	P	P	P	P
NM_000527.5(LDLR):c.1737C>G (p.Asp579Glu)	ASP579GLU	T	P	P	P	B	P	P	P	P
NM_000527.5(LDLR):c.1736A>G (p.Asp579Gly)	ASP579GLY	T	P	P	P	P	P	P	P	P
NM_000527.5(LDLR):c.1735G>T (p.Asp579Tyr)	ASP579TYR	T	P	P	P	P	P	P	P	P
NM_000527.5(LDLR):c.1736A>T (p.Asp579Val)	ASP579VAL	T	P	P	P	P	P	P	P	P
NM_000527.5(LDLR):c.1738T>C (p.Ser580Pro)	SER580PRO	T	P	P	P	P	P	P	P	P

NM_000527.5(LDLR):c.1743A>T (p.Lys581Asn)	LYS581ASN	T	P	P	P	P	B	P	P	P
NM_000527.5(LDLR):c.1744C>T (p.Leu582Phe)	LEU582PHE	V	P	B	P	P	P	P	P	P
NM_000527.5(LDLR):c.1745T>C (p.Leu582Pro)	LEU582PRO	V	P	B	P	P	P	P	P	P
NM_000527.5(LDLR):c.1748A>G (p.His583Arg)	HIS583ARG	T	P	P	P	P	P	P	P	P
NM_000527.5(LDLR):c.1747C>G (p.His583Asp)	HIS583ASP	T	P	B	P	P	P	P	P	P
NM_000527.5(LDLR):c.1749C>G (p.His583Gln)	HIS583GLN	T	P	P	P	B	P	P	P	P
NM_000527.5(LDLR):c.1754T>A (p.Ile585Asn)	ILE585ASN	T	P	P	P	P	P	P	P	P
NM_000527.5(LDLR):c.1754T>C (p.Ile585Thr)	ILE585THR	T	P	P	P	P	P	P	P	P
NM_000527.5(LDLR):c.1756T>C (p.Ser586Pro)	SER586PRO	T	P	P	P	P	P	P	P	P
NM_000527.5(LDLR):c.1775G>A (p.Gly592Glu)	GLY592GLU	T	P	P	P	P	P	P	P	P
NM_000527.5(LDLR):c.1784G>T (p.Arg595Leu)	ARG595LEU	V	P	P	P	P	B	P	P	P
NM_000527.5(LDLR):c.1783C>T (p.Arg595Trp)	ARG595TRP	T	P	P	P	P	P	P	P	P
NM_000527.5(LDLR):c.1792A>C (p.Ile598Leu)	ILE598LEU	V	B	P	P	P	P	P	P	P
NM_000527.5(LDLR):c.1796T>G (p.Leu599Trp)	LEU599TRP	T	P	P	P	B	P	P	P	P
NM_000527.5(LDLR):c.1802A>G (p.Asp601Gly)	ASP601GLY	T	B	P	B	P	B	B	P	P
NM_000527.5(LDLR):c.1801G>T (p.Asp601Tyr)	ASP601TYR	T	P	P	P	B	P	P	P	P
NM_000527.5(LDLR):c.1802A>T (p.Asp601Val)	ASP601VAL	T	P	P	P	P	P	P	P	P
NM_000527.5(LDLR):c.1808A>G (p.Lys603Arg)	LYS603ARG	T	B	P	P	P	P	P	P	P
NM_000527.5(LDLR):c.1809G>C (p.Lys603Asn)	LYS603ASN	V	B	P	P	P	P	P	P	P
NM_000527.5(LDLR):c.1814T>C (p.Leu605Pro)	LEU605PRO	T	P	P	P	P	P	P	P	P
NM_000527.5(LDLR):c.1817C>A (p.Ala606Asp)	ALA606ASP	V	P	P	P	P	P	P	P	P
NM_000527.5(LDLR):c.1820A>G (p.His607Arg)	HIS607ARG	V	P	P	P	P	P	P	P	P
NM_000527.5(LDLR):c.1823C>G (p.Pro608Arg)	PRO608ARG	T	P	P	P	P	P	P	P	P
NM_000527.5(LDLR):c.1823C>T (p.Pro608Leu)	PRO608LEU	T	P	B	P	P	P	P	P	P
NM_000527.5(LDLR):c.1822C>T (p.Pro608Ser)	PRO608SER	T	P	P	P	P	P	P	P	P
NM_000527.5(LDLR):c.1822C>A (p.Pro608Thr)	PRO608THR	T	P	P	P	P	P	P	P	P
NM_000527.5(LDLR):c.1825T>C (p.Phe609Leu)	PHE609LEU	V	B	P	P	B	P	B	B	P
NM_000527.5(LDLR):c.1829C>G (p.Ser610Cys)	SER610CYS	T	P	P	P	P	P	P	P	P
NM_000527.5(LDLR):c.1829C>T (p.Ser610Phe)	SER610PHE	T	P	P	P	P	P	P	P	P
NM_000527.5(LDLR):c.1833G>T (p.Leu611Phe)	LEU611PHE	T	P	P	P	P	P	P	P	P
NM_000527.5(LDLR):c.1834G>T (p.Ala612Ser)	ALA612SER	T	B	P	P	B	B	B	P	P

NM_000527.5(LDLR):c.1844A>T (p.Glu615Val)	GLU615VAL	V	P	P	P	B	P	P	P	P
NM_000527.5(LDLR):c.1856T>G (p.Phe619Cys)	PHE619CYS	V	P	P	P	P	P	P	P	P
NM_000527.5(LDLR):c.1855T>C (p.Phe619Leu)	PHE619LEU	V	P	P	P	P	P	P	P	P
NM_000527.5(LDLR):c.1856T>C (p.Phe619Ser)	PHE619SER	T	P	P	P	P	P	P	P	P
NM_000527.5(LDLR):c.1858T>C (p.Trp620Arg)	TRP620ARG	T	P	P	P	P	P	P	P	P
NM_000527.5(LDLR):c.1862C>G (p.Thr621Arg)	THR621ARG	T	P	P	P	P	P	P	P	P
NM_000527.5(LDLR):c.1865A>C (p.Asp622Ala)	ASP622ALA	V	P	P	P	P	P	P	P	P
NM_000527.5(LDLR):c.1864G>A (p.Asp622Asn)	ASP622ASN	T	P	P	P	P	P	P	P	P
NM_000527.5(LDLR):c.1880C>A (p.Ala627Asp)	ALA627ASP	T	P	P	P	P	P	P	P	P
NM_000527.5(LDLR):c.1879G>A (p.Ala627Thr)	ALA627THR	V	P	B	B	P	B	P	P	P
NM_000527.5(LDLR):c.1880C>T (p.Ala627Val)	ALA627VAL	T	P	P	P	P	B	P	P	P
NM_000527.5(LDLR):c.1883T>C (p.Ile628Thr)	ILE628THR	V	P	P	P	P	P	P	P	P
NM_000527.5(LDLR):c.1886T>G (p.Phe629Cys)	PHE629CYS	V	P	B	P	P	P	P	P	P
NM_000527.5(LDLR):c.1892C>A (p.Ala631Asp)	ALA631ASP	T	P	P	P	P	P	P	P	P
NM_000527.5(LDLR):c.1897C>T (p.Arg633Cys)	ARG633CYS	V	P	P	P	P	P	P	P	P
NM_000527.5(LDLR):c.1898G>T (p.Arg633Leu)	ARG633LEU	T	P	P	P	P	B	P	P	P
NM_000527.5(LDLR):c.1904C>T (p.Thr635Ile)	THR635ILE	V	P	P	P	P	B	P	P	P
NM_000527.5(LDLR):c.1906G>A (p.Gly636Ser)	GLY636SER	T	P	P	P	P	B	P	P	P
NM_000527.5(LDLR):c.1907G>T (p.Gly636Val)	GLY636VAL	T	P	P	P	P	P	P	P	P
NM_000527.5(LDLR):c.1916T>G (p.Val639Gly)	VAL639GLY	T	P	P	P	P	B	P	P	P
NM_000527.5(LDLR):c.1925T>C (p.Leu642Ser)	LEU642SER	V	P	P	P	P	P	P	P	P
NM_000527.5(LDLR):c.1937T>A (p.Leu646Gln)	LEU646GLN	V	P	P	P	P	P	P	P	P
NM_000527.5(LDLR):c.1936C>A (p.Leu646Ile)	LEU646ILE	V	P	B	P	P	P	P	P	P
NM_000527.5(LDLR):c.1943C>T (p.Ser648Phe)	SER648PHE	T	P	B	P	P	P	B	P	P
NM_000527.5(LDLR):c.1942T>C (p.Ser648Pro)	SER648PRO	V	P	B	B	P	B	P	P	P
NM_000527.5(LDLR):c.1945C>T (p.Pro649Ser)	PRO649SER	V	P	P	P	P	P	P	P	P
NM_000527.5(LDLR):c.1951G>T (p.Asp651Tyr)	ASP651TYR	T	P	P	P	P	P	P	P	P
NM_000527.5(LDLR):c.1952A>T (p.Asp651Val)	ASP651VAL	V	P	P	P	P	P	P	P	P
NM_000527.5(LDLR):c.1954A>G (p.Met652Val)	MET652VAL	V	B	B	P	P	P	B	P	P
NM_000527.5(LDLR):c.1965C>G (p.Phe655Leu)	PHE655LEU	T	P	B	B	P	B	P	P	P
NM_000527.5(LDLR):c.1968C>G (p.His656Gln)	HIS656GLN	T	P	P	P	B	P	P	P	P

NM_000527.5(LDLR):c.1973T>C (p.Leu658Pro)	LEU658PRO	V	P	P	P	P	P	P	P	P
NM_000527.5(LDLR):c.1976C>A (p.Thr659Asn)	THR659ASN	T	B	P	P	P	P	B	P	P
NM_000527.5(LDLR):c.1979A>G (p.Gln660Arg)	GLN660ARG	T	P	P	B	P	P	P	P	P
NM_000527.5(LDLR):c.1978C>A (p.Gln660Lys)	GLN660LYS	T	P	P	B	P	P	P	P	P
NM_000527.5(LDLR):c.1979A>C (p.Gln660Pro)	GLN660PRO	T	P	P	P	P	P	P	P	P
NM_000527.5(LDLR):c.1981C>G (p.Pro661Ala)	PRO661ALA	V	P	P	B	P	P	P	P	P
NM_000527.5(LDLR):c.1988G>C (p.Gly663Ala)	GLY663ALA	T	P	B	B	P	P	B	B	B
NM_000527.5(LDLR):c.1988G>A (p.Gly663Glu)	GLY663GLU	T	P	P	P	P	P	P	P	P
NM_000527.5(LDLR):c.1990G>A (p.Val664Met)	VAL664MET	T	B	P	P	P	P	B	P	P
NM_000527.5(LDLR):c.1999T>C (p.Cys667Arg)	CYS667ARG	T	P	P	P	P	P	P	P	P
NM_000527.5(LDLR):c.2000G>T (p.Cys667Phe)	CYS667PHE	T	P	P	P	P	P	P	P	P
NM_000527.5(LDLR):c.1999T>A (p.Cys667Ser)	CYS667SER	T	P	P	P	P	P	P	P	P
NM_000527.5(LDLR):c.2001T>G (p.Cys667Trp)	CYS667TRP	T	P	P	P	B	P	P	P	P
NM_000527.5(LDLR):c.2000G>A (p.Cys667Tyr)	CYS667TYR	V	P	P	P	P	P	P	P	P
NM_000527.5(LDLR):c.2026G>C (p.Gly676Arg)	GLY676ARG	T	P	P	P	P	P	P	P	P
NM_000527.5(LDLR):c.2026G>T (p.Gly676Cys)	GLY676CYS	T	P	P	P	P	P	P	P	P
NM_000527.5(LDLR):c.2029T>C (p.Cys677Arg)	CYS677ARG	V	P	P	P	P	P	P	P	P
NM_000527.5(LDLR):c.2030G>T (p.Cys677Phe)	CYS677PHE	V	P	P	P	P	P	P	P	P
NM_000527.5(LDLR):c.2030G>A (p.Cys677Tyr)	CYS677TYR	V	P	P	P	P	P	P	P	P
NM_000527.5(LDLR):c.2043C>G (p.Cys681Trp)	CYS681TRP	T	P	P	P	B	P	P	P	P
NM_000527.5(LDLR):c.2042G>A (p.Cys681Tyr)	CYS681TYR	T	P	P	P	P	P	P	P	P
NM_000527.5(LDLR):c.2045T>G (p.Leu682Arg)	LEU682ARG	V	P	P	P	P	P	P	P	P
NM_000527.5(LDLR):c.2044C>T (p.Leu682Phe)	LEU682PHE	T	P	P	P	P	P	P	P	P
NM_000527.5(LDLR):c.2045T>C (p.Leu682Pro)	LEU682PRO	T	P	P	P	P	P	P	P	P
NM_000527.5(LDLR):c.2054C>A (p.Pro685Gln)	PRO685GLN	V	P	P	P	P	P	P	P	P
NM_000527.5(LDLR):c.2054C>T (p.Pro685Leu)	PRO685LEU	T	P	P	P	P	P	P	P	P
NM_000527.5(LDLR):c.2053C>T (p.Pro685Ser)	PRO685SER	T	P	B	P	P	P	P	P	P
NM_000527.5(LDLR):c.2053C>A (p.Pro685Thr)	PRO685THR	T	P	P	P	P	P	P	P	P
NM_000527.5(LDLR):c.2056C>G (p.Gln686Glu)	GLN686GLU	V	P	P	P	P	P	P	P	P
NM_000527.5(LDLR):c.2080T>G (p.Phe694Val)	PHE694VAL	T	P	B	P	P	P	P	P	P
NM_000527.5(LDLR):c.2088C>G (p.Cys696Trp)	CYS696TRP	V	P	P	P	B	P	P	P	P

NM_000527.5(LDLR):c.2087G>A (p.Cys696Tyr)	CYS696TYR	V	P	P	P	P	P	P	P	P
NM_000527.5(LDLR):c.2089G>C (p.Ala697Pro)	ALA697PRO	T	P	P	P	P	P	P	P	P
NM_000527.5(LDLR):c.2093G>T (p.Cys698Phe)	CYS698PHE	T	P	P	P	P	P	P	P	P
NM_000527.5(LDLR):c.2093G>A (p.Cys698Tyr)	CYS698TYR	V	P	P	P	P	P	P	P	P
NM_000527.5(LDLR):c.2100C>G (p.Asp700Glu)	ASP700GLU	V	P	B	P	B	P	P	B	P
NM_000527.5(LDLR):c.2113G>T (p.Ala705Ser)	ALA705SER	T	B	P	P	B	P	B	P	P
NM_000527.5(LDLR):c.2119G>A (p.Asp707Asn)	ASP707ASN	T	P	B	P	P	B	P	P	P
NM_000527.5(LDLR):c.2120A>T (p.Asp707Val)	ASP707VAL	T	P	P	P	P	P	P	P	P
NM_000527.5(LDLR):c.2131T>G (p.Cys711Gly)	CYS711GLY	T	P	P	P	P	P	P	P	P
NM_000527.5(LDLR):c.2132G>T (p.Cys711Phe)	CYS711PHE	T	P	P	P	P	P	P	P	P
NM_000527.5(LDLR):c.2131T>A (p.Cys711Ser)	CYS711SER	T	P	P	P	P	P	P	P	P
NM_000527.5(LDLR):c.2132G>A (p.Cys711Tyr)	CYS711TYR	T	P	P	P	P	P	P	P	P
NM_000527.5(LDLR):c.2140G>C (p.Glu714Gln)	GLU714GLN	T	P	B	B	B	B	B	B	B
NM_000527.5(LDLR):c.2140G>A (p.Glu714Lys)	GLU714LYS	T	P	B	B	B	B	P	B	B
NM_000527.5(LDLR):c.2155G>C (p.Val719Leu)	VAL719LEU	T	B	P	P	P	P	B	P	P
NM_000527.5(LDLR):c.2209A>G (p.Arg737Gly)	ARG737GLY	V	B	P	P	P	P	P	P	P
NM_000527.5(LDLR):c.2224A>G (p.Thr742Ala)	THR742ALA	V	B	P	P	P	P	P	P	P
NM_000527.5(LDLR):c.2251C>T (p.Arg751Trp)	ARG751TRP	V	B	B	B	P	P	P	P	P
NM_000527.5(LDLR):c.2260G>A (p.Gly754Arg)	GLY754ARG	T	B	P	P	P	P	P	P	P
NM_000527.5(LDLR):c.2260G>T (p.Gly754Trp)	GLY754TRP	T	P	P	P	B	B	B	B	B
NM_000527.5(LDLR):c.2289G>T (p.Glu763Asp)	GLU763ASP	T	B	P	B	P	P	P	P	P
NM_000527.5(LDLR):c.2294T>G (p.Val765Gly)	VAL765GLY	T	B	P	P	P	P	B	P	P
NM_000527.5(LDLR):c.2364G>T (p.Arg788Ser)	ARG788SER	T	B	P	P	P	P	P	P	P
NM_000527.5(LDLR):c.2396T>G (p.Leu799Arg)	LEU799ARG	T	P	P	P	P	P	P	P	P
NM_000527.5(LDLR):c.2398G>A (p.Val800Ile)	VAL800ILE	V	B	P	P	B	P	P	P	P
NM_000527.5(LDLR):c.2407T>C (p.Cys803Arg)	CYS803ARG	V	P	P	B	B	P	B	P	B
NM_000527.5(LDLR):c.2413G>C (p.Gly805Arg)	GLY805ARG	T	P	P	P	P	B	P	B	P
NM_000527.5(LDLR):c.2414G>A (p.Gly805Glu)	GLY805GLU	T	P	P	P	P	P	P	B	P
NM_000527.5(LDLR):c.2417T>A (p.Val806Asp)	VAL806ASP	T	B	B	P	P	P	B	P	P
NM_000527.5(LDLR):c.2422C>G (p.Leu808Val)	LEU808VAL	V	P	B	B	P	B	P	B	B
NM_000527.5(LDLR):c.2473A>G (p.Asn825Asp)	ASN825ASP	T	P	P	P	P	P	P	P	P

NM_000527.5(LDLR):c.2475C>A (p.Asn825Lys)	ASN825LYS	V	P	P	P	P	P	P	P	P
NM_000527.5(LDLR):c.2476C>T (p.Pro826Ser)	PRO826SER	T	P	P	P	P	P	P	P	P
NM_000527.5(LDLR):c.2476C>A (p.Pro826Thr)	PRO826THR	V	P	P	P	P	P	P	P	P
NM_000527.5(LDLR):c.2479G>T (p.Val827Phe)	VAL827PHE	T	P	P	P	P	P	P	P	P
NM_000527.5(LDLR):c.2483A>G (p.Tyr828Cys)	TYR828CYS	T	P	P	P	P	P	P	P	P
NM_000527.5(LDLR):c.2482T>C (p.Tyr828His)	TYR828HIS	T	P	P	P	P	P	P	P	P
NM_000527.5(LDLR):c.2483A>C (p.Tyr828Ser)	TYR828SER	T	P	P	P	P	P	P	P	P
NM_000527.5(LDLR):c.2531G>A (p.Gly844Asp)	GLY844ASP	T	P	P	P	P	P	P	P	P
NM_000527.5(LDLR):c.2530G>A (p.Gly844Ser)	GLY844SER	V	P	P	P	P	B	P	P	P

T/V= Training or validation; P/B= Pathogenic or benign; CV= ClinVar; Poly=Polyphen-2; Mut=Mutation Taster; Opti=OptiMo-LDLr.

Table S2: Accuracy (Sn, Sp and predicted mutations) of each software and combination per each domain and the whole protein.

		MLb-LDLr		PolyPhen-2		SIFT		Mutation Taster*		REVEL		VERITY		OptiMo-LDLR	
		No.	Sn/Sp	No.	Sn/Sp	No.	Sn/Sp	No.	Sn/Sp	No.	Sn/Sp	No.	Sn/Sp	No.	Sn/Sp
Signal seq.	Pat.	1	0,25	3	0,75	4	1	2	0,67	4	1	2	0.5	4	1
	Ben.	3	1	3	1	1	0,67	3	1	2	0.66	3	1	3	1
LBD	Pat.	271	0,91	274	0,92	258	0,86	242	0,81	292	0.98	289	0.96	292	0,98
	Ben.	13	0,65	12	0,6	17	0,85	12	0,6	4	0.2	18	0.9	19	0,95
EGF-A	Pat.	20	0,95	21	1	19	0,92	19	0,92	20	0.95	20	0.95	20	0.95
	Ben.	1	1	1	1	1	1	1	1	0	0	1	100	1	100
EGF-B	Pat.	37	1	36	0,97	34	0,90	36	1	37	1	37	1	37	1
	Ben.	2	1	2	1	2	1	0	0	2	1	2	1	2	1
B-prop	Pat.	170	0,88	175	0,90	166	0,86	181	0,97	191	0.97	189	0.96	191	0,95
	Ben.	17	0,81	19	0,90	19	0,90	13	0,65	6	0.28	18	0.85	21	1
EGF-C	Pat.	34	0,92	34	0,92	30	0,81	31	1	35	0.94	33	0.89	34	0,92
	Ben.	3	1	3	1	3	1	2	0,67	0	0	3	1	3	1
O-Linked	Pat.	0	0	1	1	1	1	0	0	0	0	0	0	0	0
	Ben.	6	1	4	0,67	5	0,83	5	1	5	0.83	6	1	6	1
Trans.	Pat.	1	1	1	1	1	1	1	1	1	1	1	1	1	1
	Ben.	2	1	2	1	2	1	0	0	1	0.5	2	1	2	1
Cyt.	Pat.	11	0,79	12	0,86	13	0,93	13	0,93	13	0.92	11	0.78	12	0,86
	Ben.	2	1	2	1	0	0	2	1	1	0.5	2	1	2	1
**Total**	**Pat.**	**562**	**0,92**	**575**	**0,94**	**542**	**0,89**	**539**	**0,97**	**580**	**0.95**	**555**	**0.91**	**586**	**0.96**
	**Ben.**	**49**	**0,82**	**49**	**0,82**	**52**	**0,87**	**40**	**0,68**	**22**	**0.36**	**58**	**0.95**	**60**	**0.98**

No.=Number; Sn/Sp=Sensitivity and Specificity; Pat=Pathologic; Ben=Benign; Signal seq.=Signal Sequence; LBD=Ligand Binding Domain; EGF-A=Epidermal Growth Factor A domain; EGF-B= Epidermal Growth Factor B domain; B-prop =Beta Propeller; EGF-C=Epidermal Growth Factor C domain; Trans= Transmembrane domain; Cyt=Cytosolic domain.

Table S3: Experimental validation of the model. 93 newly *LDLR* variants classified as Benign/Likely Benign/Pathogenic/Likely Pathogenic by the ClinVar expert panel were used to validate the model. Variants with pathogenicity score above 3.5 were considered pathogenic, according to the model threshold, and those with values lower than 3.5 were considered benign.

LDLR variant	Reviewed by ClinVar expert panel	Pathogenicity score
NM_000527.5(LDLR):c.1A>T (p.Met1Leu)	Pathogenic	4.02
NM_000527.5(LDLR):c.2T>C (p.Met1Thr)	Likely pathogenic	4.59
NM_000527.5(LDLR):c.44T>C (p.Leu15Pro)	Likely pathogenic	3.23
NM_000527.5(LDLR):c.44T>G (p.Leu15Arg)	Likely pathogenic	3.80
NM_000527.5(LDLR):c.81C>G (p.Cys27Trp)	Pathogenic	6.46
NM_000527.5(LDLR):c.106G>T (p.Asp36Tyr)	Pathogenic	4.29
NM_000527.5(LDLR):c.147C>A (p.Ser49Arg)	Likely benign	1.55
NM_000527.5(LDLR):c.246C>G (p.Cys82Trp)	Likely pathogenic	6.54
NM_000527.5(LDLR):c.268G>A (p.Asp90Asn)	Pathogenic	5.10
NM_000527.5(LDLR):c.281A>T (p.Asp94Val)	Likely pathogenic	5.43
NM_000527.5(LDLR):c.302A>C (p.Glu101Ala)	Likely pathogenic	5.38
NM_000527.5(LDLR):c.392A>G (p.Asp131Gly)	Pathogenic	4.49
NM_000527.5(LDLR):c.397G>T (p.Asp133Tyr)	Likely pathogenic	5.44
NM_000527.5(LDLR):c.515A>C (p.Asp172Ala)	Likely pathogenic	5.43
NM_000527.5(LDLR):c.530C>G (p.Ser177Trp)	Likely pathogenic	5.09

NM_000527.5(LDLR):c.602A>C (p.Glu201Ala)	Likely pathogenic	5.16
NM_000527.5(LDLR):c.601G>C (p.Glu201Gln)	Likely pathogenic	4.03
NM_000527.5(LDLR):c.610T>C (p.Cys204Arg)	Pathogenic/Likely pathogenic	5.48
NM_000527.5(LDLR):c.618T>A (p.Ser206Arg)	Pathogenic	6.08
NM_000527.5(LDLR):c.619G>T (p.Gly207Cys)	Pathogenic	5.10
NM_000527.5(LDLR):c.670G>C (p.Asp224His)	Likely pathogenic	4.80
NM_000527.5(LDLR):c.679G>A (p.Asp227Asn)	Likely pathogenic	5.26
NM_000527.5(LDLR):c.680A>G (p.Asp227Gly)	Pathogenic	5.45
NM_000527.5(LDLR):c.679G>C (p.Asp227His)	Likely pathogenic	5.35
NM_000527.5(LDLR):c.718G>A (p.Glu240Lys)	Pathogenic	4.83
NM_000527.5(LDLR):c.727T>A (p.Cys243Ser)	Likely pathogenic	5.35
NM_000527.5(LDLR):c.764G>T (p.Cys255Phe)	Pathogenic	5.42
NM_000527.5(LDLR):c.768C>G (p.Asp256Glu)	Likely pathogenic	4.62
NM_000527.5(LDLR):c.797A>G (p.Asp266Gly)	Likely pathogenic	5.45
NM_000527.5(LDLR):c.796G>C (p.Asp266His)	Pathogenic/Likely pathogenic	5.31
NM_000527.5(LDLR):c.851G>A (p.Cys284Tyr)	Likely pathogenic	5.43
NM_000527.5(LDLR):c.858C>A (p.Ser286Arg)	Likely pathogenic	6.03
NM_000527.5(LDLR):c.898A>G (p.Arg300Gly)	Likely pathogenic	4.32
NM_000527.5(LDLR):c.920A>C (p.Asp307Ala)	Likely pathogenic	5.50
NM_000527.5(LDLR):c.974G>A (p.Cys325Tyr)	Likely pathogenic	5.45
NM_000527.5(LDLR):c.1003G>T (p.Gly335Cys)	Pathogenic	5.29
NM_000527.5(LDLR):c.1024G>A (p.Asp342Asn)	Benign	1.03
NM_000527.5(LDLR):c.1048C>G (p.Arg350Gly)	Likely pathogenic	3.06
NM_000527.5(LDLR):c.1049G>C (p.Arg350Pro)	Pathogenic/Likely pathogenic	3.59

NM_000527.5(LDLR):c.1060 G>A (p.Asp354Asn)	Likely pathogenic	4.84
NM_000527.5(LDLR):c.1072T >G (p.Cys358Gly)	Likely pathogenic	5.43
NM_000527.5(LDLR):c.1090T >G (p.Cys364Gly)	Likely pathogenic	5.43
NM_000527.5(LDLR):c.1129T >C (p.Cys377Arg)	Pathogenic	5.48
NM_000527.5(LDLR):c.1133A >C (p.Gln378Pro)	Likely pathogenic	4.35
NM_000527.5(LDLR):c.1166C >A (p.Thr389Lys)	Likely pathogenic	4.41
NM_000527.5(LDLR):c.1174T >A (p.Cys392Ser)	Likely pathogenic	5.35
NM_000527.5(LDLR):c.1179 G>C (p.Lys393Asn)	Likely pathogenic	4.82
NM_000527.5(LDLR):c.1178A >C (p.Lys393Thr)	Pathogenic	4.96
NM_000527.5(LDLR):c.1186 G>A (p.Gly396Ser)	Likely pathogenic	4.83
NM_000527.5(LDLR):c.1208T >C (p.Phe403Ser)	Likely pathogenic	5.25
NM_000527.5(LDLR):c.1214A >G (p.Asn405Ser)	Likely pathogenic	4.69
NM_000527.5(LDLR):c.1230 G>T (p.Arg410Ser)	Likely pathogenic	6.13
NM_000527.5(LDLR):c.1274A >T (p.Asn425Ile)	Likely pathogenic	4.01
NM_000527.5(LDLR):c.1298A >C (p.Asp433Ala)	Likely pathogenic	5.17
NM_000527.5(LDLR):c.1298A >T (p.Asp433Val)	Likely pathogenic	5.23
NM_000527.5(LDLR):c.1307T >A (p.Val436Glu)	Likely pathogenic	4.63
NM_000527.5(LDLR):c.1408A >T (p.Ser470Cys)	Likely pathogenic	2.85
NM_000527.5(LDLR):c.1432 G>A (p.Gly478Arg)	Pathogenic	5.35
NM_000527.5(LDLR):c.1433 G>T (p.Gly478Val)	Likely pathogenic	5.29
NM_000527.5(LDLR):c.1438 G>T (p.Ala480Ser)	Likely pathogenic	4.63
NM_000527.5(LDLR):c.1449 G>C (p.Trp483Cys)	Pathogenic	6.49
NM_000527.5(LDLR):c.1463T >C (p.Ile488Thr)	Likely pathogenic	5.25
NM_000527.5(LDLR):c.1470 G>T (p.Trp490Cys)	Likely pathogenic	6.67

NM_000527.5(LDLR):c.1562C>T (p.Ala521Val)	Likely pathogenic	4.22
NM_000527.5(LDLR):c.1618G>T (p.Ala540Ser)	Likely pathogenic	3.98
NM_000527.5(LDLR):c.1633G>C (p.Gly545Arg)	Likely pathogenic	4.27
NM_000527.5(LDLR):c.1634G>A (p.Gly545Glu)	Pathogenic/Likely pathogenic	4.33
NM_000527.5(LDLR):c.1645G>C (p.Gly549Arg)	Likely pathogenic	5.18
NM_000527.5(LDLR):c.1692T>G (p.Asn564Lys)	Likely pathogenic	5.17
NM_000527.5(LDLR):c.1691A>C (p.Asn564Thr)	Pathogenic	5.19
NM_000527.5(LDLR):c.1693G>C (p.Gly565Arg)	Likely pathogenic	5.34
NM_000527.5(LDLR):c.1721G>A (p.Arg574His)	Likely pathogenic	4.93
NM_000527.5(LDLR):c.1727A>G (p.Tyr576Cys)	Pathogenic/Likely pathogenic	5.37
NM_000527.5(LDLR):c.1731G>C (p.Trp577Cys)	Pathogenic/Likely pathogenic	6.66
NM_000527.5(LDLR):c.1774G>A (p.Gly592Arg)	Likely pathogenic	5.30
NM_000527.5(LDLR):c.1775G>T (p.Gly592Val)	Likely pathogenic	5.35
NM_000527.5(LDLR):c.1784G>A (p.Arg595Gln)	Pathogenic	4.92
NM_000527.5(LDLR):c.1819C>G (p.His607Asp)	Pathogenic	5.34
NM_000527.5(LDLR):c.1828T>C (p.Ser610Pro)	Likely pathogenic	4.03
NM_000527.5(LDLR):c.1860G>C (p.Trp620Cys)	Likely pathogenic	6.66
NM_000527.5(LDLR):c.1955T>C (p.Met652Thr)	Likely pathogenic	2.96
NM_000527.5(LDLR):c.2042G>C (p.Cys681Ser)	Pathogenic/Likely pathogenic	5.35
NM_000527.5(LDLR):c.2086T>G (p.Cys696Gly)	Likely pathogenic	5.43
NM_000527.5(LDLR):c.2087G>T (p.Cys696Phe)	Likely pathogenic	5.41
NM_000527.5(LDLR):c.2086T>A (p.Cys696Ser)	Likely pathogenic	5.35
NM_000527.5(LDLR):c.2095C>T (p.Pro699Ser)	Likely pathogenic	5.12
NM_000527.5(LDLR):c.2098G>A (p.Asp700Asn)	Likely pathogenic	3.59

NM_000527.5(LDLR):c.2099A>G (p.Asp700Gly)	Likely pathogenic	4.70
NM_000527.5(LDLR):c.2106G>C (p.Met702Ile)	Likely benign	2.65
NM_000527.5(LDLR):c.2114C>T (p.Ala705Val)	Likely pathogenic	3.78
NM_000527.5(LDLR):c.2177C>T (p.Thr726Ile)	Benign	1.04
NM_000527.5(LDLR):c.2231G>A (p.Arg744Gln)	Likely benign	0.48
NM_000527.5(LDLR):c.2487G>C (p.Gln829His)	Pathogenic	4.40
